# Integrating HIV pre‐exposure prophylaxis and harm reduction among men who have sex with men and transgender women to address intersecting harms associated with stimulant use: a modelling study

**DOI:** 10.1002/jia2.25495

**Published:** 2020-06-19

**Authors:** Annick Bórquez, Katherine Rich, Michael Farrell, Louisa Degenhardt, Rebecca McKetin, Lucy T. Tran, Javier Cepeda, Alfonso Silva‐Santisteban, Kelika Konda, Carlos F. Cáceres, Sherrie Kelly, Frederick L. Altice, Natasha K. Martin

**Affiliations:** ^1^ Department of Medicine University of California San Diego La Jolla CA USA; ^2^ National Drug and Alcohol Research Center University of New South Wales Sydney NSW Australia; ^3^ Yale School of Medicine Yale University New Haven CT USA; ^4^ Centro de Investigación Interdisciplinaria en Sexualidad SIDA y Sociedad Universidad Peruana Cayetano Heredia Lima Peru; ^5^ Modelling and Biostatistics Burnet Institute Melbourne VIC Australia; ^6^ Yale University Center for Interdisciplinary Research on AIDS New Haven CT USA; ^7^ Centre of Excellence in Research on AIDS Faculty of Medicine University of Malaya Kuala Lumpur Malaysia; ^8^ Population Health Sciences University of Bristol Bristol United Kingdom

**Keywords:** men who have sex with men, transgender women, stimulants, HIV pre‐exposure prophylaxis, suicide, modelling

## Abstract

**Introduction:**

Among men who have sex with men (MSM) and transgender women (TW), stimulant use is high and has been associated with an increased risk of HIV infection, suicide and cardiovascular disease (CVD) mortality. We used epidemic modelling to investigate these intersecting health harms among MSM/TW in Lima, Peru and assess whether they could be mitigated by prioritizing HIV pre‐exposure prophylaxis (PrEP) and harm reduction interventions among MSM/TW who use stimulants.

**Methods:**

We adapted a dynamic model of HIV transmission among MSM/TW in Lima to incorporate stimulant use and increased HIV risk, suicide and CVD mortality. Among 6% to 24% of MSM/TW using stimulants (mostly cocaine), we modelled an increased risk of unprotected anal sex (RR = 1.35 [95%CI: 1.17 to 1.57]) obtained from local data, and increased risk of suicide (SMR = 6.26 [95%CI: 2.84 to 13.80]) and CVD (SMR = 1.83 [95%CI: 0.39 to 8.57]) mortality associated with cocaine use based on a global systematic review. We estimated the proportion of health harms occurring among MSM/TW who use stimulants in the next year (01‐2020/01‐2021). We also investigated the 10‐year impact (01‐2020/01‐2030) of: (1) PrEP prioritization for stimulant‐using MSM/TW compared to random allocation, and (2) integrating PrEP with a theoretical intervention halving stimulant‐associated risk.

**Results:**

MSM/TW in Lima will experience high HIV incidence, suicide mortality and CVD mortality (1.6/100 py, and 0.018/100 py, 0.13/100 py respectively) in 2020. Despite stimulant using MSM/TW comprising an estimated 9.5% (95%CI: 7.8 to 11.5) of all MSM/TW, in the next year, 11% 95%CI (i.e. 2.5% to 97.5% percentile) 10% to 13%) of new HIV infections, 39% (95%CI: 18% to 60%) of suicides and 15% (95%CI: 3% to 44%) of CVD deaths could occur among this group. Scaling up PrEP among all stimulant using MSM/TW could prevent 19% (95%CI: 11% to 31%) more HIV infections over 10 years compared to random allocation. Integrating PrEP and an intervention to halve stimulant‐associated risks could reduce new HIV infections by 20% (95%CI: 10% to 37%), suicide deaths by 14% (95%CI: 5% to 27%) and CVD deaths by 3% (95%CI: 0% to 16%) over a decade.

**Conclusions:**

MSM/TW who use stimulants experience a disproportionate burden of health harms. Prioritizing PrEP based on stimulant use, in addition to sexual behaviour/gender identity criteria, could increase its impact. Integrated substance use, harm reduction, mental health and HIV care among MSM/TW is needed.

## 1 Introduction

Globally, HIV prevalence among men who have sex with men (MSM) and transgender women (TW) is 19 [1, 2] and 49‐fold [3] higher, respectively, than the general population. Substance use, and in particular stimulant use, is also higher among MSM and TW in a range of settings [4]. Understanding the role of substance use in HIV risk and other associated health harms, including overdose, accidental injuries, mental health disorders and cardiovascular diseases (CVD) [5], is key to developing an integrated public health response to multiple intersecting epidemics.

Stimulants, such as cocaine and meth/amphetamines cause euphoria, sociability and energy [5], and can increase sexual desire, pleasure, prolong sexual performance and decrease sexual inhibition and pain sensation during anal intercourse [6, 7, 8, 9]. As such, they are used by MSM and TW in a range of sexual contexts, including sex with intimate or casual partners, group sex or sex work. While causality is not well established, engagement in stimulant use and higher risk sexual behaviours are considered to co‐occur within these broader risk environments [10]. In particular, stimulant use has been associated with unprotected anal sex and with HIV infection [6, 11, 12, 13, 14, 15].

In addition to elevated HIV risks, global systematic reviews reveal a substantially elevated overall mortality among people who use cocaine or meth/amphetamines [16]. Higher suicide, overdose [17, 18, 19], CVD [20, 21], accidental injuries [22] and homicide related mortality [23], contribute to this higher overall mortality. Among MSM and TW, stimulant use has been associated with suicide ideation and attempt [24, 25, 26, 27], supporting these global findings. The lifetime prevalence of suicide attempt among MSM is estimated at between 20% and 30% (fourfold higher than among heterosexual males) [28, 29, 30, 31, 32]. Among TW, estimates vary between 25% and 51% [31, 33, 34, 35, 36]. Additionally, mounting evidence indicates an increased risk of CVD among sexual minorities, with stimulant use contributing to this excess risk [37, 38, 39, 40, 41].

While there are no approved medications to treat either cocaine or meth/amphetamine dependence [42], harm reduction can be achieved through the provision of condoms [43], HIV [44] and STI [45] pre‐exposure prophylaxis (PrEP), counselling and pharmacological treatment of mental health disorders [46, 47], and safe drug use and nutrition interventions to prevent overdose, accidental injuries and CVD.

Epidemic modelling can be used to quantify the contribution of groups to intersecting epidemics, and the potential population‐level impact of interventions, thereby guiding evidence‐based policymaking [48]. As in most Latin American countries, the HIV epidemic in Peru is concentrated among MSM and TW (13% and 27% prevalence in Lima respectively) and the country is currently considering providing HIV PrEP for these groups through the public health system. Peru is a major producer of cocaine and, in Lima, 6% to 24% of MSM/TW (varying by group) report stimulant use (mostly cocaine) in the past 3 months. We used a dynamic epidemic model to quantify the intersecting burden of stimulant use and HIV transmission, suicide and CVD mortality among MSM and TW in Lima [49], and to estimate the impact of PrEP prioritization by stimulant use and of PrEP integration with harm reduction services.

## 2 Methods

### 2.1 Mathematical model

We modified a previously published epidemic model of HIV transmission among MSM and TW in Lima, Peru [49], to incorporate stimulant use, as well as explicitly represent mortality from suicide and CVD. The model is described elsewhere [49], but in brief, it considers differences in sexual behaviours based on sexual orientation, gender identity and engagement in sex work through explicitly representing four groups: homosexual self‐identified MSM, heterosexual/bisexual self‐identified MSM, male sex workers (MSW) and TW (including those who engage in sex work). Differences in the type of sexual partners (regular, casual and commercial) and condom use and sexual positioning by type of partner among each group are represented. The model is then further disaggregated by stimulant use (yes/no), with differential prevalence of stimulant use by group, differential frequency of unprotected anal sex by stimulant use, and differential suicide and CVD mortality rates by stimulant use. Due to a lack of data on stimulant use trajectories specifically among MSM/TW, for the baseline model we assume MSM/TW enter as either using stimulants or not, and remain in these groups, but we explore turnover in a sensitivity analysis.

### 2.2 Model parameterization

The model was mainly parameterized using data from multiple local MSM/TW surveillance rounds, local epidemiological studies and global reviews and meta‐analyses. Regarding stimulant use, the 2011 surveillance round among MSM/TW is the most recent source of comprehensive drug use and sexual behaviour data among MSM/TW in Peru. Using these data, we calculated the prevalence of stimulant use in the past 3 months in each group in Lima, corresponding to 6.2% (95%CI: 4.6 to 7.8) among homosexual identified MSM, 13.3% (95%CI: 10.6 to 16.1) among heterosexual/bisexual identified MSM, 23.61% (95%CI: 20.7 to 26.5) among MSW and 17.8% (95%CI: 14.3 to 21.4) among TW. Virtually all MSM/TW (98%) who reported stimulant use were using cocaine and/or cocaine paste (see Appendix S1) and therefore we used estimates of health harms associated with cocaine use for all our analyses.

#### 2.2.1 Stimulant use and unprotected anal sex

We used the 2011 surveillance data to estimate the pooled relative risk between stimulant use and unprotected anal sex with MSM/TW using log‐binomial regression, corresponding to 1.35 (95%CI: 1.17 to 1.57) (see Appendix S1, Table S1a).

#### 2.2.2 Stimulant use and mortality associated with suicide and CVD

Estimates of suicide mortality among MSM/TW are not available and inferring these from data on suicide attempt and ideation would be highly uncertain. We therefore applied the crude mortality rate (CMR) associated with suicide among people who use cocaine obtained from a recent global systematic review [5] (0.07/100 person years (95%CI: 0.04 to 0.10)), to all MSM/TW, but explored higher rates based on suicide attempt estimates in sensitivity analyses. Among MSM/TW who do not use stimulants, we divided it by the standardized mortality ratio (SMR) for the increased risk of suicide among people who use cocaine, also obtained from the literature review (6.26 (95%CI: 2.84 to 13.80)) [5]. Similarly, due to lack of MSM/TW‐specific data, we used the CMR and SMR associated with CVD mortality among people who use cocaine from the global review [5] (CMR: 0.13/100 person years (95%CI: 0.07 to 0.24), SMR: 1.83 (95%CI: 0.39 to 8.57)) [5]. To represent uncertainty in the CMR and SMR values for suicide and CVD, we sampled from the 95% confidence interval values using the lognormal distribution.

### 2.3 Model calibration

The HIV epidemic (and mortality from suicide and CVD) was simulated with 10,000 different parameter sets randomly sampled through Latin hypercube sampling. The log likelihood of each simulated epidemic trajectory was calculated based on time series HIV prevalence data from 1985 to 2011 for the total MSM/TW population and for each group (39 data points overall), time series HIV incidence data from 1999 to 2014 for the total MSM/TW population (5 data points), the proportion using stimulants per group in 2011 and the total ART coverage for 2011 (see Appendix S1 for full detail on parameter estimates and calibration data). Fits with a log likelihood above the 99th percentile were selected for the analysis.

### 2.4 Contribution of MSM/TW who use stimulants to HIV incidence and suicide and cardio‐vascular disease mortality

We used our calibrated model fits to predict HIV incidence, prevalence and mortality associated with suicide and CVD among MSM/TW in Lima for 2020. To estimate the excess burden of HIV incidence among MSM/TW who use stimulants, we calculated the proportion of new HIV infections estimated to occur among this group in 2020 and divided it by the proportion of the total MSM/TW population who use stimulants. We similarly estimated the excess burden of suicide and CVD mortality among MSM/TW who use stimulants.

### 2.5 PrEP allocation by stimulant use

We assumed PrEP effectiveness of 80% [50, 51] across groups assuming a distribution into three adherence groups: 84% in the high, 8% in the medium and 8% in the low adherence groups (with 90%, 50% and 20% adherence respectively). In the model when a person enters PrEP, they remain on PrEP until they exit the model and new people enter PrEP (or not) at each time step based on whether the intended coverage in a specific group is achieved. We compare the impact of prioritizing PrEP to all MSM/TW who use stimulants versus allocating PrEP independently of stimulant use in each of these groups (called “random allocation” hereafter). In the PrEP prioritization scenario, we simulated 100% PrEP coverage among HIV susceptible MSM/TW who use stimulants, varying between approximately 6% and 24% depending on the group and 0% coverage among MSM/TW who do not use stimulants. In the random allocation scenario, the same proportion from each group was covered by PrEP, reproducing PrEP coverage per group in the prioritization scenario, but it was allocated proportionally among MSM/TW who use stimulants and those who do not.

Scenario 1 (PrEP prioritization by stimulant use): $\begin{array}{c} \varsigma_{k,2}\left(t\right)=1\\ \varsigma_{k,1}\left(t\right)=0 \end{array}$

Scenario 2 (Random PrEP allocation): $\varsigma_{k,f}\left(t\right)=X_{k,2}^{1}/\sum_{f}X_{k,f}^{1}$ corresponding to the proportion using stimulants in each group.

Where $\varsigma_{k,f}\left(t\right)$ is the PrEP coverage at time point (*t*) among those in sexual behaviour group *k* and stimulant use group *f* (1: no stimulant use; 2: stimulant use) and $X_{k,f}^{1}$ corresponds to the number of susceptible individuals in each sexual behaviour/stimulant use group.

### 2.6 Harm reduction intervention(s)

Due to a lack of effective treatment for stimulant use, we simulated the potential benefits of a hypothetical harm reduction intervention package assumed to reduce the excess risks of HIV, suicide and CVD mortality associated with stimulant use by half. This could be as a result of decreases in the intensity/frequency of stimulant use or through combined harm reduction interventions including condom use distribution and promotion, psychological/psychiatric treatment to prevent suicide and CVD treatment to prevent CVD mortality.

### 2.7 Sensitivity analyses

#### 2.7.1 PrEP adherence and stimulant use

While evidence is mixed [52], some studies have found lower adherence to PrEP among MSM/TW who use stimulants compared to those who do not [53, 54]. Among MSM/TW participating in the recent iPrEX open label extension study, MSM/TW with moderate to heavy cocaine use had 2.32 greater odds of having levels of tenofovir below the level of quantitation compared to MSM/TW with no cocaine use [53]. We therefore performed a sensitivity analysis using a greater proportion of stimulant users in the low adherence group (73%, 8% and 19% in the high, medium and low adherence groups, respectively, details in Appendix S1).

#### 2.7.2 Duration of stimulant use

We performed a sensitivity analysis assuming turnover between groups using and not using stimulants, with an average duration of stimulant use of 5 years.

#### 2.7.3 Suicide rates among MSM and TW

We assumed a two to sevenfold higher rate of suicide among MSM (compared to general population) and a one to threefold higher rate among TW compared to MSM, to acknowledge evidence of higher suicide rates among these populations.

#### 2.7.4 PrEP prioritization by gender identity/sexual behaviour

We implemented a scenario in which 100% of TW would be covered, with the remainder given to MSW to compare the effectiveness of this established strategy to that of PrEP prioritization by stimulant use.

## 3 Results

### 3.1 Model predictions of HIV, suicide, CVD mortality burden among MSM/TW by group

Our model estimates MSM and TW in Lima experience a high burden of HIV incidence, suicide mortality and CVD mortality, with estimates of 1.63 per 100 person‐years (/100 py) (95% Confidence Interval (2.5 and 97.5 percentiles): 0.83 to 2.51), 0.018/100 py (95%CI: 0.008 to 0.040) and 0.13/100 PY (95%CI: 0.03 to 0.5), respectively, in 2020.

**Figure 1:**
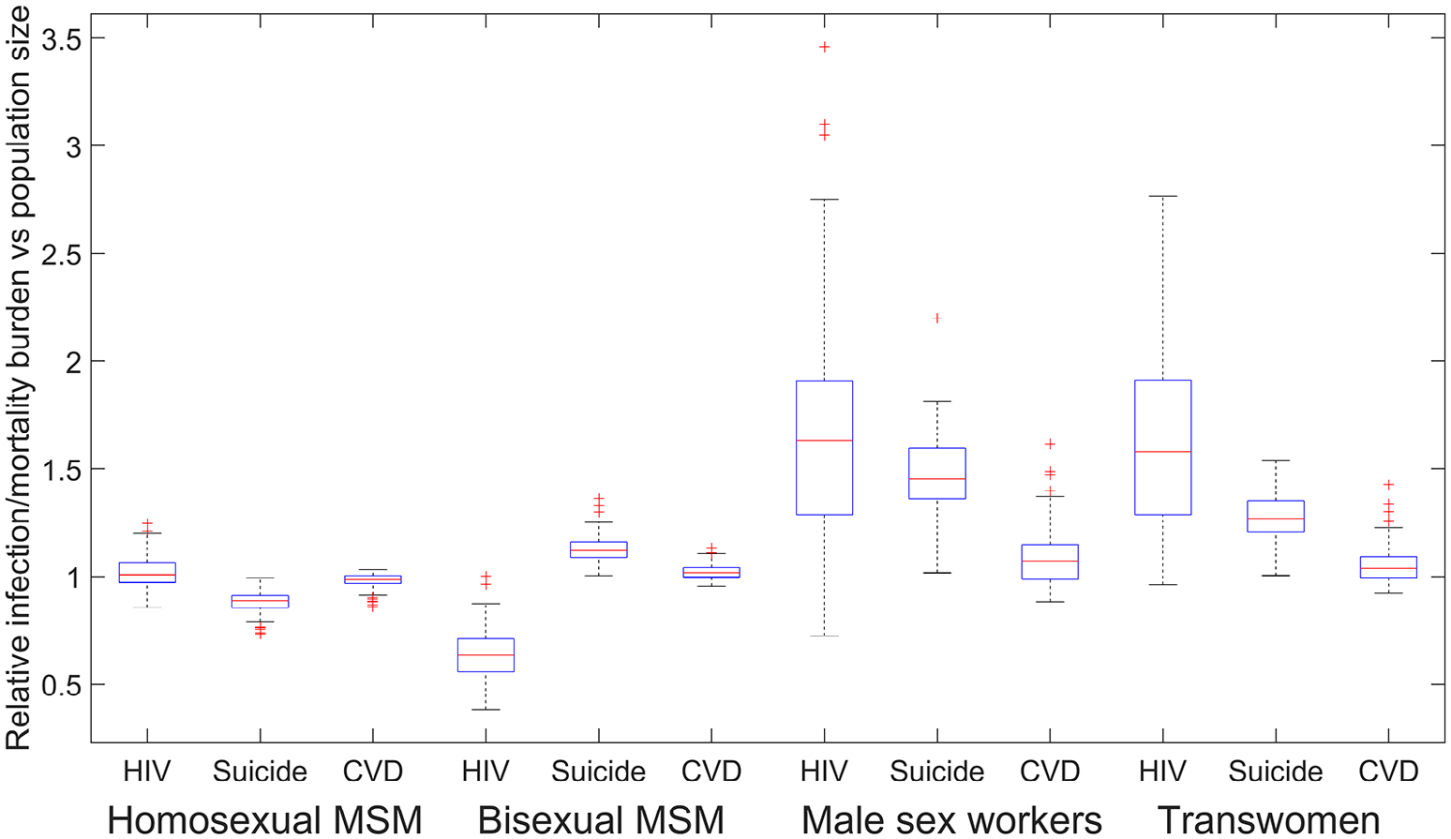
Ratio of the proportion of new HIV infections, suicide deaths and CVD deaths in each group and the proportion of the total population in each group in 2020 in Lima. A ratio of >1 indicates more than the expected proportion of infections/deaths in each group, based on population size. Red lines denote median values, boxes denote 25% to 75% confidence intervals, whiskers denote minimum and maximum values not considered as outliers, and dots denote outliers. CVD, cardiovascular disease; MSM, men who have sex with men.

The burden of each of these health harms differs by group, with 7.1% (95%CI: 4.7 to 10.0), 5.6% (95%CI: 3.6 to 8.7) and 4.7% (95%CI: 3.3 to 7.1) of new HIV infections, suicide deaths and CVD deaths, respectively, occurring among TW when they comprise 4.5% (95%CI: 2.9% to 6.7%) of the total MSM/TW population and 12.4% (95%CI: 8.7 to 16.9), 12.2% (95%CI: 4.2 to 22.9) and 9.0% (95%CI: 3.3 to 17.2) of new HIV infections, suicide deaths and CVD deaths, respectively, occurring among MSW when they comprise 8.3% (95%CI: 3.4% to 15.0%) of the total MSM/TW population. The ratios of the proportion of HIV infections, suicides and CVD deaths among each group in one year divided by the proportion of individuals in each group are shown in Figure 1, with ratios >1 indicating a disproportionate burden of infections/deaths. Our analysis indicates that MSW and TW are particularly disproportionately affected by HIV, suicide and CVD mortality.

### 3.2 Model predictions of HIV, suicide and CVD mortality burden among MSM/TW who use stimulants

**Figure 2:**
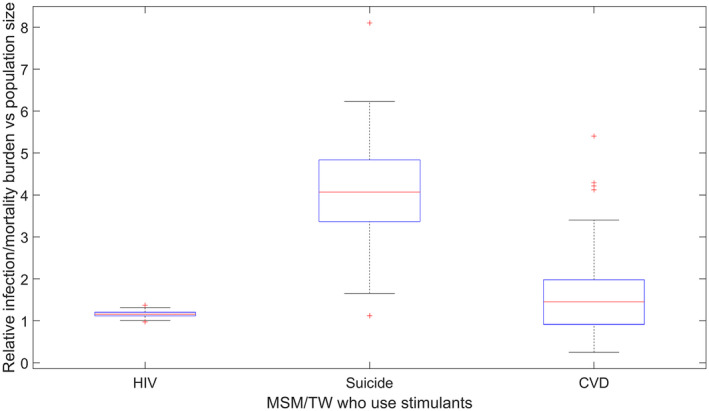
Ratio of the proportion of new HIV infections, suicide deaths and CVD deaths among MSM/TW who use stimulants and the proportion of the total population in this group in 2020 in Lima. A ratio of >1 indicates more than the expected proportion of infections/deaths among stimulant using MSM/TW, based on the population size. Lines denote median values, boxes denote 25% to 75% confidence intervals, whiskers denote minimum and maximum values not considered outliers, and dots denote outliers. CVD, cardiovascular disease; MSM, men who have sex with men; TW, transgender women.

Our modelling indicates that despite MSM/TW who use stimulants comprising an estimated 9.5% (95%CI: 7.8 to 11.5) of the overall MSM/TW population in Lima, between 2020 and 2021, 11% (95%CI: 10% to 13%) of new HIV infections, 39% (95%CI: 18% to 60%) of suicides and 15% (95%CI: 3% to 44%) of CVD deaths would occur among this group. The ratios of the proportion of HIV infections, suicides and CVD deaths among MSM/TW who use stimulants by the proportion of MSM/TW in this group are shown in Figure 2. The median ratios for all health outcomes (HIV incidence, suicide, CVD mortality) are all> 1 indicating a disproportionate burden, with some simulations for CVD < 1, reflecting the wide uncertainty interval of the SMR for CVD associated with cocaine use (which straddles 1).

### 3.3 Impact of prioritization of PrEP to MSM/TW who use stimulants

**Figure 3:**
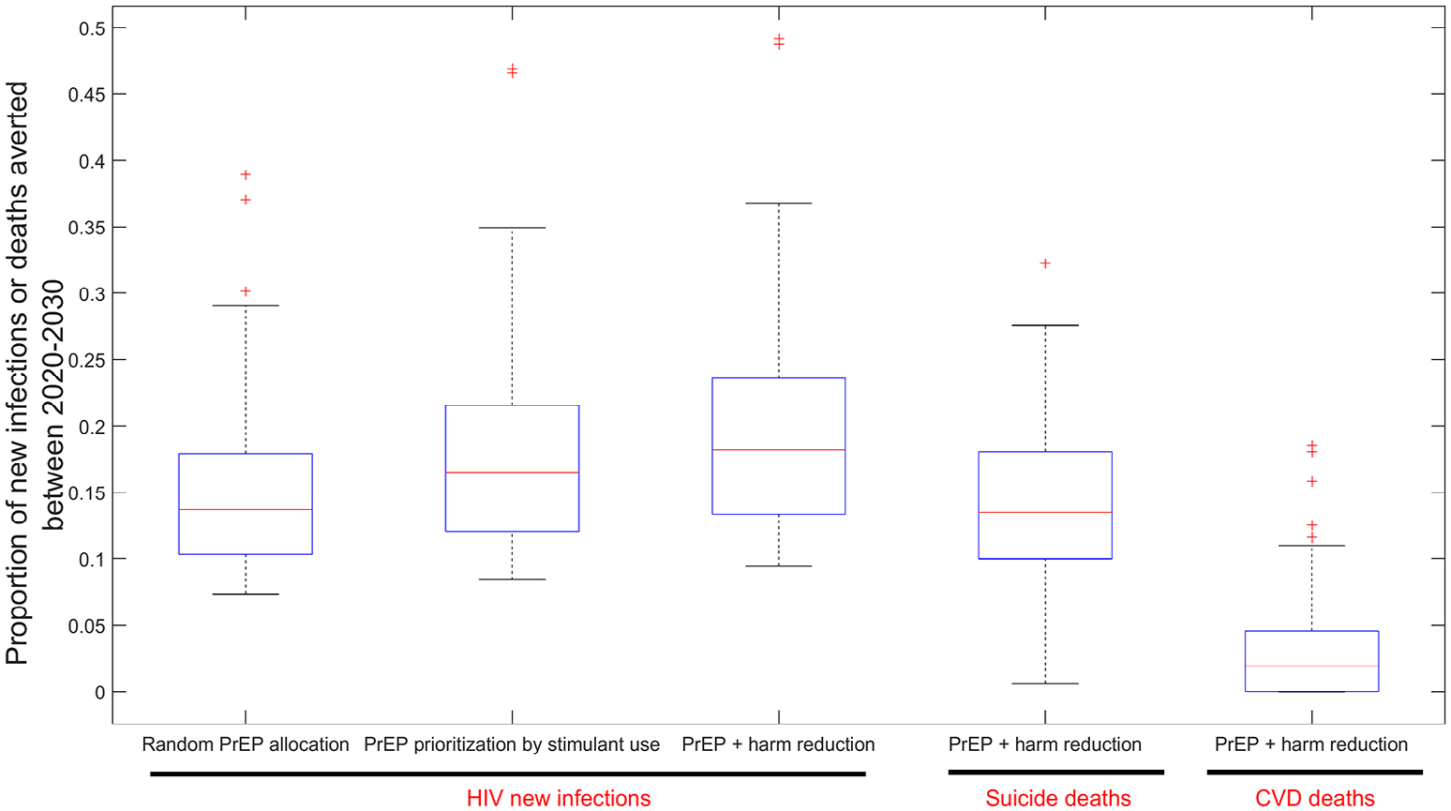
Proportion of HIV new infections, suicide and cardio‐vascular disease (CVD) deaths averted among all men who have sex with men and transgender women under the different intervention scenarios. PrEP, pre‐exposure prophylaxis.

Scaling up PrEP among all (100%) MSM/TW who use stimulants in each group between 2020 and 2030 would prevent 17.9% (95%CI: 9.1 to 34.9) of new HIV infections (Figure 3). In contrast, covering the same proportion of susceptible individuals in each group, but randomly allocating to MSM/TW who use and do not use stimulants would prevent 14.9% (95%CI: 7.4 to 30.2) of new infections across a decade. Consequently, PrEP prioritization for stimulant users could prevent 19% (95%CI: 11 to 31) more new HIV infections compared to random allocation across a decade.

### 3.4 Impact of integrated PrEP and harm reduction interventions for MSM/TW who use stimulants

Scaling up PrEP among MSM/TW who use stimulants in combination with harm reduction interventions that reduce the excess risk of unprotected anal sex, suicide mortality and CVD mortality associated with stimulant use by 50%, would avert 20% (95%CI: 10% to 37%) of new HIV infections, 14% (95%CI: 5% to 27%) of suicide deaths and 3% (95%CI: 0% to 16%) of CVD deaths between 2020 and 2030 (Figure 3). The proportion of new HIV infections, suicide deaths and CVD deaths that would occur among MSM/TW who use stimulants over 10 years in this scenario would be 5% (95%CI: 2% to 8%), 29% (95%CI: 14% to 48%) and 13% (95%CI: 3% to 32%) respectively. This indicates that while PrEP in combination with harm reduction interventions could reduce HIV incidence among MSM/TW who use stimulants to a level below that among MSM/TW who do not, a more intensive intervention would be needed to eliminate both the excess suicide and CVD mortality in this group.

### 3.5 Sensitivity analyses

In the sensitivity analyses, with lower adherence to PrEP among MSM/TW who use stimulants, prioritization of PrEP to stimulant users was still more effective compared to random allocation (11% (95%CI: 3% to 22%) more effective, compared to 19% (95%CI: 11% to 31% with no adherence differences).

Assuming a five‐year duration of stimulant use, the proportion of new HIV infections occurring among MSM/TW who use stimulants was virtually unchanged at 11.1% (95%CI: 9.7% to 13.3%) and the relative increased impact of the PrEP prioritization scenario was 17% (95%CI: 9% to 31%) under this shorter duration of stimulant use (vs. 19% (95%CI: 11% to 31%) at baseline), indicating a strategy of prioritization by stimulant use may be slightly less effective under shorter durations of stimulant use.

Assuming higher suicide rates among MSM (two to sevenfold) and TW (one to threefold higher than among MSM), translated to a suicide incidence of 0.08/100 py (95%CI: 0.02 to 0.19) versus 0.018/100 py (95%CI: 0.008 to 0.040 at baseline; with 10.9% (95%CI: 4.8% to 18.4%) of suicide deaths (vs. 5.6% (95%CI: 3.6% to 8.7%) at baseline) occurring among TW (see boxplot of relative suicide burden by group in Appendix S1).

A PrEP prioritization strategy based on gender identity/sexual behaviour led to a 22% (95%CI: 9% to 41%) reduction in new HIV infections between 2020 and 2030, which is 23% greater than obtained through the prioritization strategy based on stimulant use.

## 4 Discussion

Our study quantified the burden of HIV, suicide mortality and CVD mortality in association with stimulant use among MSM and TW in Lima, Peru. We chose Peru as a useful case study due to the high prevalence of HIV and stimulant use among MSM and TW, comparable to other global settings. We found that MSM/TW who use stimulants are disproportionately affected by incident HIV infection, suicide mortality and CVD mortality. In addition, male sex workers and TW had a higher burden of these health harms compared to homosexual and heterosexual/bisexual MSM (who do not engage in sex work) due to increased sexual risk behaviours and stimulant use. Indeed, substance use among TW in Lima has been described [55, 56] as part of the practice of street sex work, to cope with violence, hunger and long hours standing at a corner, highlighting the need to account for multiple intersecting risks within a broader context of vulnerability (see Box 1).

Box 1 **Policy Implications of integrating the HIV and harm reduction responses on HIV and stimulant use associated mortality among MSM and TW in Lima, Peru**

**Table:** 

**Assess excess risk of substance use and multiple health harms among MSM/TW beyond HIV.** In addition to their excess risk of HIV compared to the general population, MSM and TW in Lima have a higher prevalence of stimulant use and are at increased risk of multiple associated health harms including suicide and CVD mortality. Considering these health harms when assessing the health of MSM/TW would lead to pertinent referrals. **Address excess risk of multiple health harms among MSM/TW who use stimulants.** MSM and TW who use stimulants are at increased risk of multiple health harms including HIV infection, suicide and CVD mortality compared to those who do not use stimulants. Incorporating sensitive substance use screening in the context of HIV testing, prevention and care among MSM/TW to facilitate their access to harm reduction interventions could effectively reduce both HIV transmission and mortality from suicide and CVD. **Prioritize pre‐exposure prophylaxis (PrEP) among MSM/TW who use stimulants.** Given higher engagement in HIV associated risk behaviours among MSM/TW who use stimulants, prioritizing PrEP among this group could increase its impact on reducing overall HIV incidence among MSM/TW. Our modelling indicates that this would still be valid if stimulant use was associated with lower PrEP adherence, although this depends on baseline adherence levels and is therefore setting specific. **Recognize heterogeneities in HIV risk, stimulant use and associated health harms among MSM/TW.** In Lima, TW and male sex workers had both higher engagement in HIV risk behaviours and higher prevalence of stimulant use. Identifying and addressing vulnerabilities beyond HIV risk, including suicide and CVD mortality, on the basis of gender identity and engagement in sex work is important when planning the design of integrated services. Considering the broader risk environment is key.

Our modelling indicates that PrEP prioritization for MSM/TW who use stimulants could increase its impact in Lima, even with potentially lower PrEP adherence among this group. In practice, this would entail integrating substance use and sexual risk assessment within one clinical visit when prescribing PrEP, which currently does not happen systematically. More importantly, even when questions on substance use are asked, this information is rarely acted upon, meaning that referral to substance use counselling, treatment, or harm reduction strategies are not delivered. In this study, we also showed that providing harm reduction interventions in combination with PrEP among MSM/TW who use stimulants would result in substantial overall reductions in HIV, suicide and CVD mortality. Our sensitivity analysis showed that given higher HIV incidence among TW and MSW, a PrEP prioritization strategy based on gender identity/sexual behaviour would have a larger impact compared to a prioritization strategy by stimulant use, but overall our study suggests that stimulant use should be included as an additional criterion in current guidelines [57] (see Box 1). Importantly, we assume the same PrEP adherence across groups, which would likely require increased adherence support among TW, given lower adherence among this group in the IPrEX trial [58].

To our knowledge this is the first modelling study examining the impact of stimulant use among MSM/TW on HIV, suicide and CVD, but it supports recent modelling highlighting the excess risks of stimulant use on HIV and related infections among PWID [5]. The estimated relative burden of HIV incidence among MSM/TW who use stimulants (18% (95%CI: 0% to 37%) higher) was low in comparison to findings from observational studies in other settings [11], although most have focussed on meth/amphetamines, which might have a stronger effect than cocaine on sexual risk behaviours [5]. The relative risk of unprotected anal intercourse among stimulant using MSM/TW in our sample is low compared to other settings and we did not assume any other differences in sexual behaviours (for example number of partners) by stimulant use [11, 59] potentially leading to conservative estimates (see Box 2). No other modelling studies have explored PrEP prioritization by stimulant use, but findings will vary between settings depending on baseline levels of adherence and therefore similar modelling exercises should be undertaken to inform local decision making (see Box 2).

Box 2 **Research Agenda of Integrating HIV prevention and harm reduction services in Peru**

**Table:** 

Our data indicate stimulant use is associated with unprotected anal sex. Further modelling analyses should incorporate associations between stimulant use and other HIV risk behaviours, including number of sexual partners, frequency of sex and contact with partners at higher risk of HIV to more comprehensively represent HIV risk associated with stimulant use. Globally, disaggregated data by sexual orientation and gender identity are missing for most health outcomes including suicide and CVD mortality. Given evidence on higher prevalence of major depressive episodes and suicide ideation and attempt among MSM and TW in particular, research that quantifies excess suicide mortality among these populations is needed. Increased risk of suicide and CVD among HIV‐positive individuals has been documented in some settings. Further modelling analyses should evaluate the impact of such patterns on CVD and suicide mortality rates among HIV‐positive MSM/TW in Peru. Other health outcomes associated with stimulant use and with gender identity or sexual orientation such as depression, psychosis, sexually transmitted infections, fatal accidental injuries and violence, were not explored in this analysis and warrant inclusion in modelling studies in order to provide a complete picture of multiple intersecting health harms in this population. When data are available, applying individual based modelling approaches could better represent risk heterogeneities for multiple health harms. PrEP scale up is at an early stage in Peru and despite the potential benefits of prioritizing it to MSM/TW who use stimulants, reaching and retaining them in PrEP might present challenges. Further research is needed to better understand PrEP engagement patterns among MSM/TW in Peru. While there is no proven effective treatment to reduce stimulant use, multiple interventions are available to reduce harms associated with stimulant use. Explicitly modelling these different intervention packages and associated costs would allow to identify cost effective strategies to inform the implementation of integrated services for MSM/TW in Peru. The feasibility of providing integrated health services among MSM/TW in Peru, that address sexual health, mental health and substance use will need to be assessed in order to identify barriers and devise solutions.

Our study has limitations. First, like all models ours was limited by data uncertainties. For example we lacked MSM/TW and setting‐specific data on suicide and CVD mortality overall and by stimulant use. As a result, our model might underestimate overall infection/mortality burden and dilute differences between groups. While we implemented a sensitivity analysis to address this in relation to suicide rates, the study should be updated when MSM/TW‐specific data become available (see Box 2). Importantly, the latest available HIV surveillance data among MSM/TW in Lima is from 2011. A new surveillance round was implemented in 2019, but findings are not yet published, and the sampling methodology differed from previous rounds, affecting comparability. In addition, a public sector PrEP demonstration study (i.e. ImPrEP) is ongoing in Peru and will provide empirical data to strengthen modelling to benefit prioritization strategies. Due to a lack of sexual network data, we use a compartmental model that does not fully represent the sexual network structure and its potential effect on both HIV transmission and intervention impact.

Second, we simulated a theoretical intervention package as there are no effective pharmacotherapies for stimulant use, and psychosocial therapies have weak or nonspecific effects. Our study therefore presents the potential impact if a stimulant use treatment were developed, or if a package of interventions were provided to address HIV, suicide and CVD among MSM/TW who use stimulants. Studies have shown low to moderate effectiveness of psychosocial interventions including motivational interviewing, contingency management and cognitive behavioural therapy, on reducing both methamphetamine use and risky sexual behaviours among MSM [42]. Among TW, hormonal therapy, body enhancement modifications and sexual reassignment surgeries (when needed) improves psychological outcomes [60]. A recent review recommended incorporating community‐based and harm reduction interventions to these modalities to enhance impact [61]. During the past years, the Ministry of Health in Peru has sought to move mental health from specialized services towards primary care and community‐based services, passing a new Mental Health Law in 2019 [62]. Mental health providers have been trained to focus on the intersection between substance use and sexual orientation/gender identity, aiming to reach MSM and TW [63]. Decreased access to healthcare across public services [64] and among MSM/TW [65] may impair success, so involving community‐based organizations in intervention delivery would help address some of these issues (See Box 1).

Third, while our model incorporates much complexity in terms of sexual behaviours and stimulant use by sexual orientation, gender identity and engagement in sex work; a full range of intersecting health risks exists that we did not explore. Associations between HIV infection and suicidality have been consistently reported. Similarly, there is evidence for higher risk of CVD among HIV‐positive patients [66, 67, 68]. Additionally, a range of nonfatal harms are associated with both stimulant use and HIV, including increased incidence of STI and depression [69, 70]. Further epidemic modelling of the impact of stimulant use and these intersecting risks among key populations at risk of HIV is warranted (see Box 2).

## 5 Conclusions

Our modelling indicates that prioritization of HIV PrEP among MSM/TW in Lima who use stimulants could enhance PrEP prioritization strategies based on sexual behaviours, or sexual orientation/gender identify. Stronger integration of interventions that address stimulant use among MSM/TW in HIV programmes is key to reducing multiple associated harms among this population. Importantly, these interventions should be complementary to broader interventions addressing the vulnerabilities and structural gaps that affect the wellbeing of sexual minorities in Peru. Interventions to guarantee their fundamental rights, including the right to an official identification reflecting their gender, the right to protection from harassment and violence, and the right to employment and health, are key to reducing persistent health disparities.

## Competing interests

NM has received unrestricted research grants from Gilead and Merck unrelated to this work. All other authors declare no competing interests.

## Authors’ contributions

AB and NM conceptualized the study analyses and wrote the first draft of the manuscript. AB developed the model and generated model results and figures. LT, LD, RM, SK and MF conducted the systematic reviews and meta‐analyses to inform model parameterization. RA provided data to inform the model and provided guidance in its analyses and interpretation of results. KR and KK conducted statistical analyses to inform the model parameterization. CC, AS and JC aided in manuscript drafting. All authors critically reviewed and approved the manuscript for submission.

## Funding

This article as part of the *Integrating services for HIV and related comorbidities: modelling to inform policy and practice* Supplement was supported by the US National Institutes of Health, Fogarty International Center. Additional support was provided by the National Institute on Drug Abuse (DP2DA049295 [AB], K01DA043421 [JC], R01DA037773 [NM], R01DA1104470 [LD]), the National Institute of Allergy and Infectious Diseases (R01AI147490 [NM]), the Australian National Drug and Alcohol Research Center [MF], the National Health and Medical Research Fellowship [LD], the Curtin Senior Research Fellowship [RM], UNITAID through the ImPrEP study [CC] and [KK].

## Supporting information

Appendix S1. Model specifications, equations, parameterization and analyses.
