# Supplementary material for: Integrating HIV pre‐exposure prophylaxis and harm reduction among men who have sex with men and transgender women to address intersecting harms associated with stimulant use: a modelling study
Source: J Int AIDS Soc. 2020 Jun 19;23(Suppl 1):e25495. doi: 10.1002/jia2.25495 (PMC7305413; doi:10.1002/jia2.25495)
Supplement: Supplementary file 1 — Appendix S1. Model specifications, equations, parameterization and analyses. [file JIA2-23-e25495-s001.docx]

**Technical appendix**

**Integrating HIV pre-exposure prophylaxis and harm reduction among men who have sex with men and transgender women to address intersecting harms associated with stimulant use: a modelling study**

Borquez A, Rich K, Farrell M, Degenhardt L, McKetin R, Tran L, Cepeda J, Silva-Santisteban A, Konda K, Cáceres CF, Kelly S, Altice R, Martin NK.

Prevalence of stimulant use is relatively high among men who have sex with men (MSM) and transgender women (TW) in a range of settings, including in Lima, Peru, where cocaine is the most commonly used stimulant. As stimulant use has been associated with higher risk sexual behaviours and higher risk of suicide and cardiovascular disease (CVD) mortality among MSM/TW, we investigated the burden of both harms among MSM/TW in Lima using mathematical modelling. We modified a published mathematical model[^1^](#_ENREF_1) of HIV transmission among MSM/TW in Lima to represent stimulant use and associated risks for HIV transmission and suicide and CVD mortality. We also used the model to investigate the impact of prioritizing HIV pre-exposure prophylaxis among MSM/TW who use stimulants versus random allocation by stimulant use and of combining PrEP to a harm reduction intervention that would halve stimulant associated risks. In this technical appendix, we describe (1) the technical specification of the deterministic, compartmental model of sexual HIV transmission and suicide among MSM/TW, (2) the parameter values and sources for Lima, Peru (3) the model fitting procedure.

### Technical specification of the MSM/TW model

Full detail of the original model has been published,[^1^](#_ENREF_1) but we provide an updated description of its specifications, including the representation of stimulant use and suicide mortality as well as updated data sources.

#### Definition of MSM/TW groups

To represent HIV spread in the model, we defined four interacting groups of MSM/TW: men who have sex with men and women (MSMW) and generally identify as heterosexual or bisexual, men who mostly have sex with men (MMSM) and generally identify as homosexual/gay, male sex workers, corresponding to men who reported having anal sex with men in exchange for money, drugs, gifts or favours in the past 12 months, and TW at higher risk, including those who engage in sex work. These categories are intended to represent a broad spectrum of gender identities, sexual orientations and risk contexts which are associated with sexual behaviours including numbers of partners, types of partnerships formed (stable, casual, commercial), condom use, and sexual positioning.

#### Stimulant use and HIV risk

A study using data from the Peruvian 2002-2003 HIV surveillance round among MSM/TW found a significant positive association between HIV infection and cocaine use before or during sex. The 2011 surveillance round among MSM/TW is the most recent source of comprehensive sexual behaviour data among MSM/TW in Peru, with a total sample of 5575 across all sites in Peru and 3182 in Lima. Using these 2011 data, associations between stimulant use (cocaine, cocaine paste, poppers, amphetamines) and sexual risk behaviours, including high number of partners, unprotected sex and sex work were found, suggesting HIV risk is higher among MSM/TW who use stimulants and that this has persisted through time. We used the Lima sample from 2011 to calculate prevalence of stimulant use in the past 3 months in each group and we estimated the pooled relative risk between stimulant use and unprotected anal sex with MSM/TW using log-binomial regression (**Table S1a**). Detail of the distribution by type of stimulant used is shown in **Table S1b**.

Variables were defined as follows:

**Stimulant use**: having used poppers, ecstasy, cocaine, cocaine paste and/or amphetamines in the past 3 months

**Unprotected sex**: Unprotected receptive or insertive anal sex at last sex with a man or TW in the past 3 months

##### Table S1a. Prevalence of stimulant use and associated risk of unprotected sex among MSM/TW in Lima, Peru in 2011

|  | **Prevalence of stimulant use in past 3 months (95%CI)** |
| --- | --- |
| Men who mostly have sex with men (MMSM) N=852 | 6.22% (4.6-7.8) |
| Men who mostly have sex with women (MMSW) N=593 | 13.32% (10.6-16.1) |
| Male sex workers (MSW) N=847 | 23.61% (20.7-26.5) |
| Transgender women (TW) N=449 | 17.82% (14.3-21.4) |
|  | **Association between stimulant use past 3 months and NO condom use** |
| Relative risk of condom use at last sex with a man or transgender woman among MSM/TW who use stimulants versus not | RR 1.35 (95%CI 1.17-1.57) p<0.0001 |

**Table S1b. Distribution of MSM/TW in Lima, Peru, who reported stimulant use by drug type.**

|  | Number |
| --- | --- |
| Cocaine Only | 143 |
| Cocaine + Pasta Basica de Cocaine | 146 |
| Cocaine + Pasta Basica de Cocaine + Amphetamine | 9 |
| Cocaine + Pasta Basica de Cocaine + Ecstasy | 7 |
| Cocaine + Pasta Basica de Cocaine + Ecstasy + Amphetamine | 21 |
| Cocaine + Amphetamine | 1 |
| Cocaine + Ecstasy | 10 |
| Cocaine + Amphetamine + Ecstasy | 1 |
| Amphetamine | 3 |
| Amphetamine + Ecstasy | 2 |
| Ecstasy | 3 |
| Pasta Basica + Amphetamine | 1 |
| Pasta Basica + Ecstasy | 3 |
| Pasta Basica + Amphetamine + Ecstasy | 0 |
| Pasta Basica only | 62 |
|  |  |
| TOTAL | 412 |

We further disaggregated the population in the epidemic model in two groups by stimulant use in the past 3 months (yes/no) and incorporated the increased risk of unprotected sex among MSM/TW who use stimulants in the force of infection calculation (further detail and equations provided in section **Force of infection**).

Little is known about the average duration of stimulant use among MSM and TW, but studies indicate that use can be chronic with some individuals reporting use for over 20 years, while for others, use is sporadic over shorter periods of time. Given drug use is more common among the MSM/TW communities and that the populations represented here, and particularly MSW and TW, experience a range of social vulnerabilities, we assumed stimulant use to be constant while sexually active with other MSM/TW. A fixed proportion in each group were therefore assumed to use stimulants at entry into the model and remained in this state until they exited the population. We performed a sensitivity analysis assuming an average duration of stimulant use of 5 years in order to investigate the impact of this assumption on our model findings.

#### Stimulant use and suicide

While high rates of suicidal ideation and attempt among MSM/TW have been reported in Peru, estimates of suicide mortality among MSM/TW are not available (and these are difficult to obtain across settings). We therefore applied the crude mortality rate (CMR) associated to suicide among people who use stimulants obtained from the literature review[^2^](#_ENREF_2) (0.07/100 person years (0.04, 0.10)) to all MSM/TW. Among MSM/TW who do not use stimulants, we divided it by the standard mortality ratio (SMR) for the increased risk of suicide among people who use stimulants, also obtained from the literature review (6.26 (2.84, 13.80)). To represent uncertainty in these values, we sampled from the 95% confidence interval of the CMR and the SMR using the lognormal distribution.

#### Stimulant use and cardiovascular disease morality

We applied the crude mortality rate (CMR) associated to CVD among people who use stimulants obtained from the literature review (0.13/ 100person years (0.07-0.24) to all MSM/TW. Among MSM/TW who do not use stimulants, we divided it by the standard mortality ratio (SMR) for the increased risk of CVD mortality among people who use stimulants, also obtained from the literature review (1.83 (0.39-8.57)). To represent uncertainty in these values, we sampled from the 95% confidence interval of the CMR and the SMR using the lognormal distribution.

#### HIV progression and treatment

The course of HIV infection was represented as distinct phases of disease progression defined by duration and infectiousness (**Figure S1**)[^3^](#_ENREF_3). When an individual gets infected, they enter a phase of acute infection (short duration, high infectiousness) and progresses to a latent phase with a CD4 cell count >350 cells/mm^3^ and to a latent phase with a CD4 cell count<350-200 cells/mm^3^ (both have long duration, low infectiousness), before entering a pre-AIDS phase with a CD4 cell count<200 cells/mm^3^ (short duration, high infectiousness). The disease finally progresses to an AIDS phase (short duration, no infectiousness due to an interruption of sexual activity), followed by death. HIV positive individuals receive antiretroviral treatment (ART) during the latent or pre-AIDS stage at a rate that varies by stage. ART reduces infectiousness and extends survival. The earlier the initiation of ART, the longer the increase in life expectancy.

##### Figure S1 – Progression of HIV infection in MSM/TW model

**
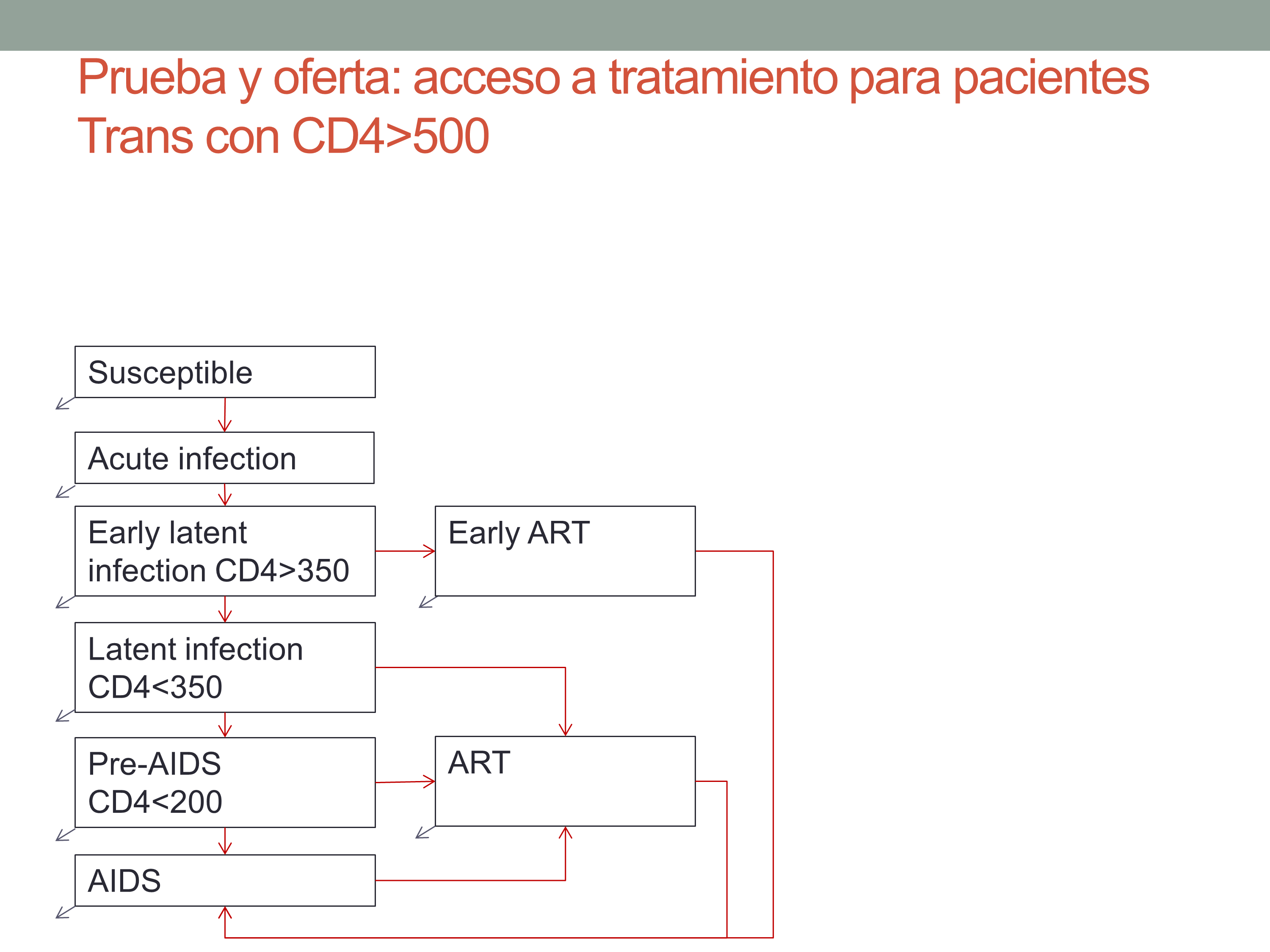
**

MSM/TW initially enter the model as susceptible and spend an average time period in the population, corresponding to the average duration of their sexual activity with MSM/TW. Homosexual and bisexual men have a slower turnover than TW at higher risk and male sex workers, which reflects the lower life expectancy among TW and the transitional aspect of sex work. Once individuals exit the model, they are replaced and allocated to each group according to the initial population distribution by group. The population grows at an average rate of 2%.[^4^](#_ENREF_4) Condom use was modelled to increase linearly from 1995 until 2005, and to remain constant at the rate reported thereafter.[^5^](#_ENREF_5) ART was introduced free of charge in Peru in 2004.[^6^](#_ENREF_6) To account for the fact that ART started being provided to some people before this policy change, we modelled its introduction with a small coverage starting in 2002 and a linear increase up to reach a coverage of 80% of HIV positive individuals with <200 CD4 cells/mm^3^ (preAIDS stage) in 2007.[^7^](#_ENREF_7) In 2012 national guidelines changed recommending treatment to HIV positive individuals with 200-350 CD4 cells/mm^3^. Guidelines changed again in December 2015 to include patients with a CD4 count <500 cells/mm^3^ and treatment independent of CD4 count was adopted in Peru in March 2018. We modelled a slow linear increase in coverage among those in the (late) latent infection, pre-AIDS and AIDS stages between 2002 and 2012 and fitted treatment rates to the estimated coverage among MSM/TW in 2011.[^8^](#_ENREF_8) We modelled a faster increase from 2012 to reflect the expansion of treatment guidelines and introduced treatment among those in early latent infection in 2016 to reflect the expansion of treatment guidelines in 2016 and 2018. These changes in guidelines have not resulted in a substantial increase in ART treatment access among MSM/TW, but we assumed coverage would keep increasing in the future at a constant rate.

#### Model equations

The model is defined by ordinary differential equations to simulate how HIV spreads over time. They are shown below. The state variables are given by$X_{k,f,a}^{s}(t)$ and $XP_{k,f,a}^{s}(t)$, corresponding to people who are not on PrEP and to people either on PrEP (while susceptible) or who have been on PrEP (if infected), respectively. *t* is the time elapsed in the simulation; *s* is the infection-status (1= susceptible; 2= acute infection; 3= latent infection; 4= pre-AIDS; 5= AIDS; 6= ART; 7=late ART), *k* is ‘sexual behaviour’ (1= Insertive MSMW; 2=Versatile MSMW; 3=Insertive MMSM; 4=Versatile MMSM; 5=Receptive MMSM; 6=Insertive sex worker; 7=Versatile sex worker; 8=Receptive sex worker; 9= receptive TW at a higher risk), f is ‘stimulant use’ (1=no; 2=yes), *a* is the PrEP adherence group (1=good; 2=average; 3=poor).

$$\begin{matrix} \frac{dX_{k,f}^{1}(t)}{dt}=\gamma_{k,a,f}\left\{\left(\mu+\alpha\left(t\right)\right)\sum_{k,f,a}^{s} \left(X_{k}^{s}+XP_{k,f,a}^{s}\right)+\sigma_{5}\sum_{k,f,a} \left(X_{k,f}^{5}+XP_{k,f,a}^{5}\right)+\sum_{k} \left(\tau_{k}\sum_{a,f}^{s} \left(X_{k,f}^{s}+XP_{k,f,a}^{s}\right)\right)+\sum_{f} \left(\left(\varpi_{f}+o_{f}\right)\sum_{k,a}^{s} \left(X_{k,f}^{s}+XP_{k,f,a}^{s}\right)\right)\right\} \\ -\lambda_{k,f}X_{k,f}^{1}-(\mu+\tau_{k}+\varpi_{f}+o_{f})X_{k,f}^{1} \\ \frac{dX_{k,f}^{2}(t)}{dt}=\lambda_{k,f}X_{k,f}^{1}-\left(\sigma_{2}+\mu+\tau_{k}+\varpi_{f}+o_{f}\right)X_{k,f}^{2} \\ \frac{dX_{k,f}^{3}(t)}{dt}=\sigma_{2}X_{k,f}^{2}-\left(\sigma_{3}+\delta\left(t\right)+\mu+\tau_{k}+\varpi_{f}+o_{f}\right)X_{k,f}^{3} \\ \frac{dX_{k,f}^{4}(t)}{dt}=\sigma_{3}X_{k,f}^{3}-\left(\sigma_{4}+\vartheta\left(t\right)+\mu+\tau_{k}+\varpi_{f}+o_{f}\right)X_{k,f}^{4} \\ \frac{dX_{k,f}^{5}(t)}{dt}=\sigma_{4}X_{k,f}^{4}+\sigma_{6}X_{k,f}^{6}+\sigma_{7}X_{k,f}^{7}-\left(\sigma_{5}+\mu+\tau_{k}+\varpi_{f}+o_{f}\right)X_{k,f}^{5} \\ \frac{dX_{k}^{6}(t)}{dt}=\delta\left(t\right)X_{k,f}^{3}-\left(\sigma_{6}+\mu+\tau_{k}+\varpi_{f}+o_{f}\right)X_{k,f}^{6} \\ \frac{dX_{k}^{7}(t)}{dt}=\vartheta\left(t\right)X_{k,f}^{4}-\left(\sigma_{7}+\mu+\tau_{k}+\varpi_{f}+o_{f}\right)X_{k,f}^{7} \end{matrix}$$

$$\begin{matrix} \text{Susceptible on PrEP and infected while they were on PrEP :} \\ \frac{dXP_{k,f,a}^{1}(t)}{dt}=-\lambda P_{k,a,f}XP_{k,f,a}^{1}-(\mu+\tau_{k}+\varpi_{f}+o_{f})XP_{k,f,a}^{1} \\ \frac{dXP_{k,f,a}^{2}(t)}{dt}=\lambda_{k,a,f}XP_{k,f,a}^{1}-\left(\sigma_{2}+\mu+\tau_{k}+\varpi_{f}+o_{f}\right)XP_{k,f,a}^{2} \\ \frac{dXP_{k,f,a}^{3}(t)}{dt}=\sigma_{2}XP_{k,f,a}^{2}-\left(\sigma_{3}+\delta\left(t\right)+\mu+\tau_{k}+\varpi_{f}+o_{f}\right)XP_{k,f,a}^{3} \\ \frac{dXP_{k,f,a}^{4}(t)}{dt}=\sigma_{3}XP_{k,f,a}^{3}-\left(\sigma_{4}+\vartheta\left(t\right)+\mu+\tau_{k}+\varpi_{f}+o_{f}\right)XP_{k,f,a}^{4} \\ \frac{dXP_{k,f,a}^{5}(t)}{dt}=\sigma_{4}XP_{k,f,a}^{4}+\sigma_{6}XP_{k,f,a}^{6}-\left(\sigma_{5}+\mu+\tau_{k}+\varpi_{f}+o_{f}\right)XP_{k,f,a}^{5} \\ \frac{dXP_{k,f,a}^{6}(t)}{dt}=\delta\left(t\right)XP_{k,f,a}^{3}-\left(\sigma_{6}+\mu+\tau_{k}+\varpi_{f}+o_{f}\right)XP_{k,f,a}^{6} \\ \frac{dXP_{k,f,a}^{7}(t)}{dt}=\vartheta\left(t\right)XP_{k,f,a}^{4}-\left(\sigma_{7}+\mu+\tau_{k}+\varpi_{f}+o_{f}\right)XP_{k,f,a}^{7} \end{matrix}$$

$$\begin{matrix} \\ \text{Flow to PrEP} \\ X_{k,f}^{1}\to X_{k,f}^{1}-\varsigma_{\text{k,f}}(t)\sum_{a} \left(X_{k,f}^{1}+XP_{k,f,a}^{1}\right)-\sum_{a} XP_{k,f,a}^{1} \\ XP_{k,f,a}^{1}\to XP_{k,f,a}^{1}+\upsilon_{a}\left(\varsigma_{\text{k}}(t)\sum_{a} \left(X_{k,f}^{1}+XP_{k,f,a}^{1}\right)-\sum_{a} XP_{k,f,a}^{1}\right) \end{matrix}$$

$1/\sigma_{s}$is the mean time spent in infection phase *s*; $\lambda_{k,f}$and$\lambda_{k,a,f}$ correspond to the force of infection for individuals in each group, depending on their sexual identity/practices, stimulant use and PrEP adherence if on PrEP; $\mu$is the death rate, $\varpi_{f}$is the rate of suicide by stimulant use, $o_{f}$is the rate of CVD mortality by stimulant use,$\frac{1}{\tau_{k}}$ is the net mean time spent in each sexual behaviour group. $\alpha\left(t\right)$is the population growth rate over time; $\gamma_{k,a,f}$ is the proportion of people by sexual behaviour, PrEP adherence and stimulant use. A certain proportion of infected individuals receive early ($\delta\left(t\right)$) or late ($\vartheta\left(t\right)$) ART depending on coverage at time t. People who exited the population due to background death, suicide, AIDS related death or population turnover are continuously replaced into the susceptible group $X^{1}$and distributed according to$\gamma_{k,a,f}$. Equally, susceptible individuals who are not on PrEP continuously move to the “susceptible on PrEP” compartment ($XP_{k,f,a}^{1}(t)$) so that the PrEP coverage at that time point $\varsigma_{k,f}\left(t\right)$ is maintained. They are distributed into each adherence group according to$\upsilon_{a}$.

#### PrEP allocation by stimulant use

We investigated two scenarios to compare the effectiveness of prioritizing PrEP to all MSM/TW who use stimulants versus allocating PrEP independently of stimulant use in each of these groups (called “random allocation” hereafter). In the first scenario we simulated 100% PrEP coverage among HIV susceptible MSM/TW who use stimulants, varying between approximately 6% and 24% depending on the group and 0% coverage among MSM/TW who do not use stimulants. In the second scenario, the same proportion of each group is covered by PrEP, reproducing PrEP coverage per group in the prioritization scenario, but it is allocated proportionally among MSM/TW who use stimulants and those who do not. These two scenarios allow us to evaluate the relative effectiveness of PrEP prioritization to MSM/TW who use stimulants.

Scenario 1 (PrEP prioritization by stimulant use): $\begin{matrix} \varsigma_{k,2}\left(t\right)=1 \\ \varsigma_{k,1}\left(t\right)=0 \end{matrix}$

Scenario 2 (Random PrEP allocation): $\varsigma_{k,f}\left(t\right)=\frac{X_{k,2}^{1}}{\sum_{f} X_{k,f}^{1}}$as obtained from scenario 1. In this way, the proportion receiving PrEP in each group is the same as in scenario 1, but the allocation is done randomly in regards to stimulant use.

The boundary conditions of the system are:

$$\begin{matrix} X_{k,a,f}^{1}(0)=(1-seed)\gamma_{k,a,f}N_{0} \\ X_{k,a,f}^{3}(0)=(seed)\gamma_{k,a,f}N_{0} \end{matrix}$$

$N_{0}$is the size of the MSM and TW population at the start of the simulation and $seed$ is the HIV prevalence at the start of the simulation in all parts of the population.

#### Force of infection

The force of infection determines the rate of progression from susceptible to infected. The force of infection through sexual contact depends on: the number of insertive and receptive partnerships $C^{i}$and $C^{r}$, respectively, the pattern of sexual partnership formation with respect to sexual behaviour (i.e. the proportion of partnerships formed with each of the sexual behaviour groups for insertive and receptive partnerships ($\rho_{k,k',f,f'}^{i}$ and$\rho_{k,k',f,f'}^{r}$ respectively by stimulant use status), the number of sex acts occurring within that partnership ($\omega_{k,k'}$), the infection-status and stage of infection of the partner, the fraction of sex acts in which a condom is used by sexual behaviour group and stimulant status ($\varphi_{k,k',f,f'}$) and the efficacy of condoms in reducing the risk of HIV transmission ($\psi$).$\beta_{s}^{i}$and $\beta_{s}^{r}$ are the probabilities of HIV transmission per sex act for each stage of infection for insertive and receptive anal sex respectively. For individuals using PrEP, it will also depend on their adherence to the PrEP regimen, which determines the proportion of sex acts protected by PrEP ($\eta_{a}$) and on the efficacy of PrEP ($\theta$). Functional PrEP effectiveness (based on adherence patterns and efficacy) was assumed to be 80% based on the PROUD[^9^](#_ENREF_9) and Ipergay[^10^](#_ENREF_10) trials, although it may be lower in Peru and among specific populations such as TW. This simplification was made to increase generalizability of our findings in the context of the review.

The frequency of condom use in partnerships is specific to each sexual behaviour group and is determined by the receptive partner. The increased risk of unprotected sex among those using stimulants was implemented through a relative risk. In partnerships in which either one or both of the partners used stimulants, condom use was reduced by the corresponding relative risk.

#### Mixing matrix

The mixing matrix designs the proportion of partnerships that are formed with individuals from each of the groups. The first step is to determine who has sex with whom. Since our groups are divided in insertive, receptive and versatile roles, there are certain combinations that cannot occur: two insertive individuals. To balance the number of insertive and receptive partnerships we first defined a mixing matrix for the insertive individuals, reflecting their preference for certain types of partners, as well as a random mixing matrix where insertive individuals chose their partner according to the proportion of sex acts offered by each of the receptive groups - this assumes that the amount of sex the “providers” have is totally dependent of the demand. The extent to which the mixing was closer to satisfy the preferences of insertive individuals was determined by a parameter$\varepsilon$ varying from one (totally assortative mixing) to zero (totally random mixing). The total number of partners among the receptive groups was then recalculated to respond to the demand and the mixing matrix for receptive individuals was updated.

$$\rho_{k,k'}^{i}=\left(\varepsilon A_{k,k'}+(1-\varepsilon)B_{k,k'}\right)$$

$$B_{k,k'}=\frac{C_{k'}^{r}\left(\sum_{s} X_{k',1}^{s}+\sum_{a,s} X_{k',a}^{s}\right)}{\sum_{k'} C_{k'}^{r}\left(\sum_{s} X_{k',1}^{s}+\sum_{a,s} X_{k',a}^{s}\right)}$$

$$\rho_{k,k',f,f'}^{i}=\rho_{k,k'}^{i}\theta_{f'}$$

$$\rho_{k,k',f,f'}^{r}=\rho_{k,k'}^{r}\theta_{f'}$$

$\rho_{k,k'}^{i}\text{ and }\rho_{k,k'}^{r}$define the proportion of total partnerships in group k that are formed with group k’ for insertive and receptive partnerships respectively. $A_{k,k'}$is the expected proportion of partnerships that insertive individuals in group k will have with receptive individuals in group k’ and $B_{k,k'}$is the proportion of receptive partnerships given by receptive individuals in group k’ out of the total number of partnerships $C^{r}$ given by all receptive individuals in all groups. $\varepsilon$determines the extent to which the mixing is in accordance to the insertive partners preferences or dependent on availability. is the updated number of receptive partnerships individuals in group k give in order to respond to the demand. $\rho_{k,k',f,f'}^{i}$and $\rho_{k,k',f,f'}^{r}$define the proportion of total partnerships in group k and f that are formed with group k’ and f’, for insertive and receptive partnerships respectively. It assumes that mixing in function of stimulant use occurs randomly (i.e. the proportion of partnerships occurring with MSM/TW who use stimulants is equal to the proportion of MSM/TW who use stimulants).

### Model parameterisation: values and sources

#### Demography

Parameters that describe the basic demography of the population are provided in **Table S2.**

##### Table S2 – Basic demography of the population

| **Parameters** | **Description** | **Value** | **Reference** |
| --- | --- | --- | --- |
| Population 15 to 49y | 2007 | 4,767,148 | [^4^](#_ENREF_4) |
| Population 15 to 49y | 1981 | 2,503,140 | [^11^](#_ENREF_11) |
| First report of AIDS cases in Peru |  | 1983 | [^12^](#_ENREF_12) |
| Proportion of the population that are men in Lima, Peru | 2007 | 0·489 | [^4^](#_ENREF_4) |
| Proportion of male-to-male sex in general population |  | 0·06 | [^13-15^](#_ENREF_13) |

#### HIV progression and treatment

Parameters that define the natural history of HIV (**Table S3**) were estimated from the global scientific literature. For reasons of computational efficiency, the uncertainty in these parameter values is not reflected in the estimates of uncertainty, with the exception of$\beta^{3-4}$ which corresponds to the baseline HIV transmission probability during an insertive anal sex act in the latent phase of infection.

##### Table S3 – Natural history of HIV model parameters

| **Parameters** | **Description** | **Symbol** | **Prior Value mode [min-max]** | **Prior Value mean[variance]** | **Posterior Value mean[variance]** | **Reference** |
| --- | --- | --- | --- | --- | --- | --- |
| Average transmission rate of HIV per sex act | if latent HIV infection | *β^3-4^* | 0·0028[0·002-0·004] | 0.0026[3.8 10-7] | 0.0029[1.5x10^-7^] | [^16^](#_ENREF_16) |
| Start of the epidemic | year |  | [1970-1982] | 1976[12] | 1972[6.9] | [^12^](#_ENREF_12) |
| Relative transmission rate per insertive anal sex act |  |  | 1 |  |  | Def. |
| Relative transmission rate per receptive anal sex act | (ref. insertive anal sex) | *ξ* | 5 |  |  | [^17^](#_ENREF_17),[^18^](#_ENREF_18) |
| Relative infectiousness in acute phase infection | (ref. latent infection) | *β^2^* | 27 |  |  | [^3^](#_ENREF_3) |
| Relative infectiousness in latent phase infection |  | *β^3-4^* | 1 |  |  | [Def.](file:///C:\aborquez\AppData\Local\Microsoft\Windows\Temporary%20Internet%20Files\Content.MSO\19CD7C14.xls#RANGE!_ENREF_1) |
| Relative infectiousness in pre-AIDS phase infection | (ref. latent infection) | *β^5^* | 7·2 |  |  | [^3^](#_ENREF_3) |
| Relative infectiousness in AIDS phase infection | (ref. latent infection) | *β^6^* | 0 |  |  | [^3^](#_ENREF_3) |
| Relative infectiousness of virally-suppressed individuals on ART | (ref. latent infection) | *β^7-8^* | 0·2 |  |  | [^3^](#_ENREF_3),[^19^](#_ENREF_19) |
| Mean duration of acute phase infection | months | *1/Ϭ_2_* | 3 |  |  | [^3^](#_ENREF_3),[^19^](#_ENREF_19) |
| Mean duration of latent phase | years | *1/ Ϭ _3_* | 10 |  |  | [^3^](#_ENREF_3),[^19^](#_ENREF_19) |
| Mean interval with elevated viral load, pre-AIDS | months | *1/ Ϭ _4_* | 10 |  |  | [^3^](#_ENREF_3),[^19^](#_ENREF_19) |
| Mean interval with AIDS before death | months | *1/ Ϭ _5_* | 9 |  |  | [^3^](#_ENREF_3),[^19^](#_ENREF_19) |
| Mean duration on ART among those starting early (latent) | years | *1/ Ϭ _6_* | 25 |  |  |  |
| Mean duration on ART among those starting late (pre-AIDS) | years | *1/ Ϭ _7_* | 12 |  |  |  |
| Mean ART initiation rate among those in latent, pre-AIDS, AIDS | 2002-2012 (slow)  2012-2030 (faster) | *ART_covlate* | 0.05-0·15 |  | 0.10[8.6x10^-4^] | [^7^](#_ENREF_7),[^8^](#_ENREF_8),[^20^](#_ENREF_20) |
| Mean ART initiation rate among those in the early latent stage | 2016-2030 | *ART_cov* | 0.05-0.15 |  | 0.10 [9.5x10^-4^] | Assumption |
| Efficacy of condoms |  | *Ψ* | 0.7 |  |  | [^21^](#_ENREF_21),[^22^](#_ENREF_22) |

##### **Table S4 – Distribution of risk in the population and risk behaviours**

| **Parameter** | **Prior Value Mode [min-max]** | **Prior Value Mean [variance]** | **Posterior Value Mean [variance]** | **Reference** |
| --- | --- | --- | --- | --- |
| Proportion of all MSM/TW: MSMW | 0·2 [0·08-0·3] | 0.19[0.002] | 0.18 [0.002] | [^5^](#_ENREF_5),[^13^](#_ENREF_13),[^23-25^](#_ENREF_23) |
| Proportion of all MSM/TW: sex worker | 0·15 [0·1-0·25] | 0.17[0.001] | 0.18 [0.001] | [^5^](#_ENREF_5),[^23^](#_ENREF_23),[^25-28^](#_ENREF_25) |
| Proportion of all MSM/TW: Transwomen | 0·05 [0·04-0·07] | 0.06[0.0002] | 0.05[0.0001] | [^23^](#_ENREF_23) |
| Mean duration: MSMW | 20-40 | 27 [8.3] | 27 [8.0] | Assumption |
| Mean duration: MMSM | 20-40 | 27[8.3] | 26[8.1] | Assumption |
| Mean duration: sex worker | 2-20 | 4[5] | 5[4.5] | Assumption |
| Mean duration: Transwomen | 10-30 | 15[8.3] | 15[7.6] | Assumption |
| N sex acts per commercial partnership | 1-2 | 1.5[0.08] | 1.7[0.05] | Assumption |
| N sex acts per stable partnership | 20-50 | 35[57] | 29 [50] | [^29^](#_ENREF_29) |
| N sex acts per casual partnership | 3-15 | 9[12] | 8 [9] | [^30^](#_ENREF_30) |
| Condom change | 2.5-3.5 | 3[0.02] | 3.1[0.07] | [^5^](#_ENREF_5),[^23^](#_ENREF_23),[^31^](#_ENREF_31),[^32^](#_ENREF_32) |
| Epsi (mixing matrix) | 0.6[0.4-0.99] | 0-66[0.015] | 0.64[0.013] | Assumption |
| *Sexual behaviour: MSMW* |  |  |  |  |
| Pr of all MSMW: insertive | 0·85 [0·8-0·9] | 0.85[0.0004] | 0.85[0.0005] | [^33^](#_ENREF_33) |
| N partnerships/year: MSMW insertive | 2[1-4] | 2.3[0.39] | 2.0[0.42] | [^31^](#_ENREF_31),[^34^](#_ENREF_34) |
| Pr of protected sex acts: MSMW insertive | 0·10-0·17 | 0.13[0.0002] | 0.13[0.0004] | [^30^](#_ENREF_30),[^33^](#_ENREF_33),[^34^](#_ENREF_34) |
| N partnerships/year: MSMW versatile | 3·5 [1.5-4] | 3[0.29] | 3 [0.29] | [^30^](#_ENREF_30),[^31^](#_ENREF_31),[^33^](#_ENREF_33),[^34^](#_ENREF_34) |
| Pr of protected sex acts: MSMW versatile | 0·10-0·17 | 0.13[0.002] | 0.14[0.0004] | [^30^](#_ENREF_30),[^34^](#_ENREF_34) |
| *Sexual behaviour: MMSM* |  |  |  |  |
| Pr of all MMSM: insertive | 0·25 [0·25-0·35] | 0.25[0.0004] | 0.25[0.0005] | [^24^](#_ENREF_24),[^28^](#_ENREF_28) |
| N partnerships/year: MMSM insertive | 1·2 [1-3] | 1.7[0.20] | 1.4[0.08] | [^31^](#_ENREF_31),[^35^](#_ENREF_35) |
| Pr of protected sex acts: MMSM insertive | 0·10-0·17 | 0.13[0.002] | 0.135[0.0004] | [^34^](#_ENREF_34) |
| Pr of all MMSM: receptive | 0·325 [0·3-0·35] | 0.33[0.0001] | 0.32[0.0001] | [^24^](#_ENREF_24),[^28^](#_ENREF_28) |
| N partnerships/year: MMSM receptive | 3[1-4] | 2.7[0.4] | 2.6[0.35] | [^31^](#_ENREF_31),[^34^](#_ENREF_34) |
| Pr of protected sex acts: MMSM receptive | 0·10-0·18 | 0.15[0.002] | 0.15[0.0004] | [^34^](#_ENREF_34) |
| N partnerships/year: MMSM versatile | 3.5 [1.5-4] | 3[0.29] | 2.9[0.35] | [^31^](#_ENREF_31),[^34^](#_ENREF_34) |
| Pr of protected sex acts: MMSM versatile | 0·10-0·18 | 0.15[0.002] | 0.15[0.0003] | [^34^](#_ENREF_34) |
| *Sexual behaviour: sex worker* |  |  |  |  |
| Pr of all sex worker: insertive | 0·20 [0·20-0·30] | 0.2[0.0004] | 0.2[0.0004] | [^35^](#_ENREF_35) |
| N partnerships/year: sex worker insertive | 30 [10-35] | 25[29] | 27[21] | [^35^](#_ENREF_35) |
| Pr of protected sex acts: sex worker insertive | 0·10-0·17 | 0.13[0.002] | 0.13[0.0004] | [^35^](#_ENREF_35) |
| Pr of all sex worker: receptive | 0·4 [0·35-0·5] | 0.42[0.001] | 0.42[0.001] | [^35^](#_ENREF_35) |
| N partnerships/year: sex worker receptive | 30 [10-50] | 30[67] | 29[23] | [^35^](#_ENREF_35) |
| Pr of protected sex acts: sex worker receptive | 0·10-0·17 | 0.13[0.001] | 0.13[0.0004] | [^35^](#_ENREF_35) |
| N partnerships/year: sex worker versatile | 50 [35-60] | 48[39] | 50[64] | [^35^](#_ENREF_35) |
| Pr of protected sex acts: sex worker versatile | 0·1-0·25 | 0.13[0.002] | 0.13[0.0003] | [^35^](#_ENREF_35) |
| *Sexual behaviour: transgender women* |  |  |  |  |
| N partnerships/year: transgender women | 80 [60-150] | 97[439] | 97[395] | [^30^](#_ENREF_30),[^31^](#_ENREF_31) |
| Pr of protected sex acts: transgender women | 0·10-0·16 | 0.13[0.001] | 0.39[0.0003] | [^34^](#_ENREF_34) |

N: number; Pr: proportion; min: minimum; max: maximum; ref: reference.

#### Contribution of each sexual behaviour/identity group to HIV incidence and suicide and CVD deaths

To estimate the excess burden of HIV incidence among each group we calculated the proportion of new HIV infections that are estimated to occur among each group in the next year (between 2020 and 2021) and calculated the excess incidence burden among this group by dividing the proportion of new infections in this group by the proportion of the total MSM/TW population who belong to this sexual behaviour/identity group.

Similarly, to estimate the excess burden of suicide and CVD mortality among each group we calculated the proportion of suicides/CVD deaths that are estimated to occur among this group in the next year (between 2020 and 2021) and divided it by the proportion of the total MSM/TW population who use stimulants.

#### Contribution of MSM/TW who use stimulants to HIV incidence and suicide and CVD deaths

To estimate the excess burden of HIV incidence among MSM/TW who use stimulants we calculated the proportion of new HIV infections that are estimated to occur among this group in the next year (between 2020 and 2021) and calculated the excess incidence burden among this group by dividing the proportion of new infections in this group by the proportion of the total MSM/TW population who use stimulants.

Similarly, to estimate the excess burden of suicide and CVD mortality among MSM/TW who use stimulants we calculated the proportion of suicides/CVD deaths that are estimated to occur among this group in the next year (between 2020 and 2021) and divided it by the proportion of the total MSM/TW population who use stimulants.

## Model fit: methods and data

The epidemic was simulated with 10,000 different parameter sets randomly sampled through Latin hypercube sampling. The log likelihood of each epidemic trajectory was calculated based in time series HIV prevalence data for the total MSM/TW population and for each group (**Table S5**), time series HIV incidence data for the total MSM/TW population (**Table S6**), the proportion using stimulants per group in 2011 (**Table S1**) and the total ART coverage for 2011 based on Chow et al.[^8^](#_ENREF_8) The fits with a log likelihood above the 99^th^ percentile were selected for the analysis.

The parameters allowed to vary were those describing sexual behaviour as well as a couple describing the natural history of infection. Descriptive statistics for their prior and posterior distributions are given in **Tables S3** and **3** when applicable. The parameters for which the posterior distributions diverged the most from the prior distribution were the basic transmission probability (towards higher values) and the year at which the epidemic started (towards earlier start) as well as the sex workers turnover (towards slower), the number of sex acts in a commercial partnership (towards higher values) and the number of sex acts in a stable partnership (towards lower values). In general, the selection process favoured parameter values that increased risk among sex workers and transwomen and decreased risk among MMSW.

### **Table S5. HIV prevalence by group in Lima, Peru**

| **Population** | **Year** | **Sample size** | **Prevalence** | **Reference** |
| --- | --- | --- | --- | --- |
| All MSM/TW | 1985 | 98 | 0·112 | [^36^](#_ENREF_36) |
| All MSM/TW | 1988 | 124 | 0·065 | [^37^](#_ENREF_37) |
| All MSM/TW | 1990 | 4300 | 0·262 | [^38^](#_ENREF_38) |
| All MSM/TW | 1996 | 444 | 0·185 | [^5^](#_ENREF_5) |
| All MSM/TW | 1997 | 1328 | 0·16 | [^39^](#_ENREF_39) |
| All MSM/TW | 1998 | 1211 | 0·178 | [^5^](#_ENREF_5) |
| All MSM/TW | 2000 | 1357 | 0·197 | [^5^](#_ENREF_5) |
| All MSM/TW | 2002 | 1358 | 0·223 | [^5^](#_ENREF_5) |
| All MSM/TW | 2006* | 618 | 0.151 |  |
| All MSM/TW | 2007 | 559 | 0·222 | [^28^](#_ENREF_28) |
| All MSM/TW | 2008 | 318 | 0·179 | [^31^](#_ENREF_31) |
| All MSM/TW | 2011* | 2171 | 0.163 | [^40^](#_ENREF_40) |
| MSMW | 1996 | 129 | 0·139 | [^5^](#_ENREF_5),[^34^](#_ENREF_34) |
| MSMW | 1998 | 263 | 0·091 | [^5^](#_ENREF_5) |
| MSMW | 2000 | 533 | 0·084 | [^5^](#_ENREF_5) |
| MSMW | 2002 | 511 | 0·129 | [^5^](#_ENREF_5) |
| MSMW | 2006* | 185 | 0.097 |  |
| MSMW | 2008 | 109 | 0·055 | [^41^](#_ENREF_41) |
| MSMW | 2008 | 21 | 0·29 | [^31^](#_ENREF_31) |
| MSMW | 2011* | 283 | 0.05 | [^40^](#_ENREF_40) |
| MMSM | 1996 | 265 | 0·18 | [^5^](#_ENREF_5),[^34^](#_ENREF_34) |
| MMSM | 1998 | 796 | 0·181 | [^5^](#_ENREF_5) |
| MMSM | 2000 | 661 | 0·26 | [^5^](#_ENREF_5) |
| MMSM | 2002 | 562 | 0·262 | [^5^](#_ENREF_5) |
| MMSM | 2006* | 261 | 0.177 |  |
| MMSM | 2008 | 253 | 0·186 | [^31^](#_ENREF_31) |
| MMSM | 2011* | 761 | 0.183 | [^40^](#_ENREF_40) |
| Transgender women | 1996 | 48 | 0·333 | [^5^](#_ENREF_5),[^34^](#_ENREF_34) |
| Transgender women | 1998 | 134 | 0·343 | [^5^](#_ENREF_5) |
| Transgender women | 2000 | 96 | 0·448 | [^5^](#_ENREF_5) |
| Transgender women | 2002 | 255 | 0·322 | [^5^](#_ENREF_5) |
| Transgender women | 2006* | 95 | 0.242 |  |
| Transgender women | 2008 | 208 | 0·178 | [^31^](#_ENREF_31) |
| Transgender women | 2009 | 459 | 0·296 | [^32^](#_ENREF_32) |
| Transgender women | 2011* | 368 | 0.264 | [^40^](#_ENREF_40) |
| Male sex workers | 2006* | 77 | 0.083 |  |
| Male sex workers | 2007 | 169 | 0·243 | [^41^](#_ENREF_41) |
| Male sex workers | 2008 | 391 | 0·207 | [^31^](#_ENREF_31) |
| Male sex workers | 2011* | 759 | 0.135 | [^40^](#_ENREF_40) |

*The eligibility criteria for the 2006 and 2011 sentinel surveillance rounds excluded HIV positive MSM/TW who were aware of their status. We corrected these estimates based on the proportion of HIV positive MSM/TW who knew their status in a study preceding the 2011 round.

### Table S6. HIV incidence (infections/100 person years)

| **Year** | **Incidence** | **Reference** |
| --- | --- | --- |
| 1999 | 3·5 [2.3-4.7] | ALASKA cohort[^25^](#_ENREF_25) |
| 2002.5 | 5.3 [1.64-9.05] | BED, 8 neighbourhoods[^42^](#_ENREF_42) |
| 2006 | 3.2 [1.0-5.35] | BED sentinel surveillance[^41^](#_ENREF_41),[^43^](#_ENREF_43) |
| 2011 | 3.6 | Comunidades positivas cohort[^41^](#_ENREF_41),[^44^](#_ENREF_44) |
| 2014 | 0.9 | SABES cohort[^41^](#_ENREF_41),[^45^](#_ENREF_45) |

### Figure S2 – Fits against prevalence data for four sub-groups and the overall MSM/TW population.

**
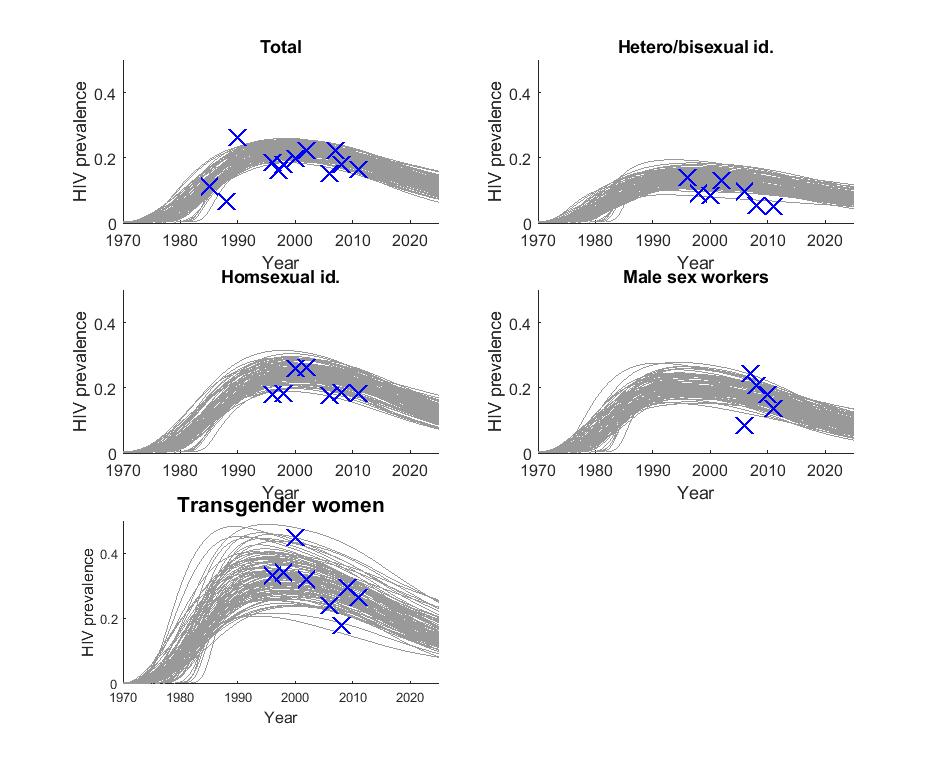
**

### Alternative PrEP scenario

We also investigated a third extreme scenario is which the total PrEP coverage would be the same as in the scenarios above but with allocation being completely proportional across groups (independently of sexual identity/behaviours or stimulant use).

$$\varsigma_{k,f}\left(t\right)=\frac{\sum_{k} X_{k,2}^{1}}{\sum_{k,f} X_{k,f}^{1}}$$

This resulted in approximately 10% PrEP coverage in each group, proportionately distributed among MSM/TW who use and do not use stimulants. The proportion of new infections averted in this scenario was 12.2% (6.5-23.3) translating in a relative effectiveness of 45%(20-71) compared to the full prioritization among MSM/TW who use stimulants. This highlights the association between prevalence of stimulant use and HIV risk behaviours, suggesting prioritization by stimulant use might also translate in prioritization by a range of other risk factors, although this will be context specific. A comparison of the three scenarios illustrates the incremental effectiveness of prioritizing PrEP by risk and by stimulant use.

### Sensitivity analyses

Duration of stimulant use. We implemented a sensitivity analysis to investigate the impact of assumptions about the duration of stimulant use. In our main analysis we assumed a lifetime duration of cocaine use, with a constant proportion of MSM/TW entering the model engaging in stimulant use. In our sensitivity analysis, we assumed the same constant proportion of MSM/TW using stimulants entering the model, but assumed an average duration of stimulant use of 5 years. The progression rate from non-stimulant use to stimulant use was calibrated to reproduce the same prevalence of stimulant use by group in each of the selected epidemic fits used in the main analysis (the Matlab lsqnonlin solver was used to determine this rate).

The results for the proportion of new HIV infections and suicide deaths occurring among MSM/TW who use stimulants was virtually unchanged at 11.1% (9.7-13.3) and 39.1% (17.6-60.0), respectively. Similarly, the proportion of HIV infections averted under the PrEP prioritization by stimulant use scenario was very similar to the baseline analysis (17.5% (9.0-34.0) versus 17.9% (9.1-34.9), respectively), and so was the impact of the random PrEP allocation scenario (15.0% (7.4-30.2) vs respectively). The relative increased impact of the PrEP prioritization scenario was 17% (9-31) under this shorter duration of stimulant use, indicating a strategy of prioritization by stimulant use may be slightly less effective under short durations of stimulant use, although the difference is small.

Lower PrEP adherence among MSM/TW who use stimulants. While evidence is mixed,[^46^](#_ENREF_46) some studies have found lower adherence to PrEP among MSM/TW who use stimulants compared to those who do not.[^47^](#_ENREF_47),[^48^](#_ENREF_48) We used findings from the most recent study assessing this question among MSM/TW participating in the IPrEX open label extension study to inform our sensitivity analysis.[^47^](#_ENREF_47) Cocaine use was measured in scalp hair samples and categorized as light (500-3000 pg/mg) and moderate to heavy (>3000 pg/mg). PrEP adherence in the first 3 months was measured through plasma tenofovir concentrations. Light cocaine use was associated with 2.10 (95% confidence interval: 1.07 to 4.14) greater odds of having levels of tenofovir below the level of quantitation and the odds were 2.32 (95%CI 1.08 to 5.00) greater among participants with moderate to heavy use, compared to no cocaine use. We used the higher odds ratio and translated it into a relative risk based on the following equation:

RR=OR/((1-p)+(pxOR)), where OR corresponds to the odds ratio for low adherence among MSM/TW who use stimulants and p corresponds to the proportion of MSM/TW who used stimulants in the IPrEX OLE sub-study (100 out of 400 participants).

RR=2.32/((1-0.25)+(0.25*2.32))

RR=1.74

In our main analysis, 84% were assumed to be in the high adherence group, 8% in the medium adherence group and 8% in the low adherence group. In our sensitivity analysis, we assumed that 16%*1.74=27% of MSM/TW who use stimulants would be in the medium or low adherence groups (versus 16% in the main analysis). This resulted in 73%, 8% and 19% of MSM/TW using stimulants in the high, medium and low adherence a group (based on the conservative assumption that all those with undetectable tenofovir had low adherence). Under these assumptions, 16.4% (8.3-32.0) of new HIV infections were averted in the PrEP prioritization by stimulant use scenario and 14.7% (7.3-29.6) under random PrEP allocation. This translated into 11% (3-22) more infections averted under through prioritization by stimulant use versus 19% (11-31) in our baseline scenario assuming no differences in adherence. These findings indicate that while lower adherence to PrEP among MSM/TW who use stimulants might substantially decrease the relative effectiveness of a program prioritizing MSM/TW who use stimulants, the strategy would still likely prevent a higher number of new infections. These findings will vary depending on baseline levels of adherence between settings and therefore similar modelling exercises should be undertaken to inform decision making in terms of PrEP allocation by stimulant use and implementation of PrEP adherence support programmes for MSM/TW who use stimulants.

**Higher suicide rates among MSM and TW.** We assumed a two to seven-fold higher rate of suicide among MSM (compared to general population) and a one to three-fold higher rate among TW compared to MSM, to acknowledge evidence of higher suicide rates among these populations. This required disaggregating the crude suicide mortality rate by sexual behaviour group and applying a different relative suicide mortality risk among all MSM (including MSW) and among TW, which was randomly sampled assuming a uniform distribution. This translated to a suicide incidence of 0.08/100py (95%I: 0.02-0.19) versus 0.018/100py (95%CI: 0.008-0.040 at baseline; with 10.9% (95%I: 4.8-18.4) of suicide deaths (versus 5.6% (95%I: 3.6-8.7) at baseline) occurring among TW. The contribution of stimulant using MSM/TW to suicide deaths and the impact of the combined PrEP and harm reduction intervention remained unchanged given the higher suicide rates were applied to stimulant using and not using groups alike.

**
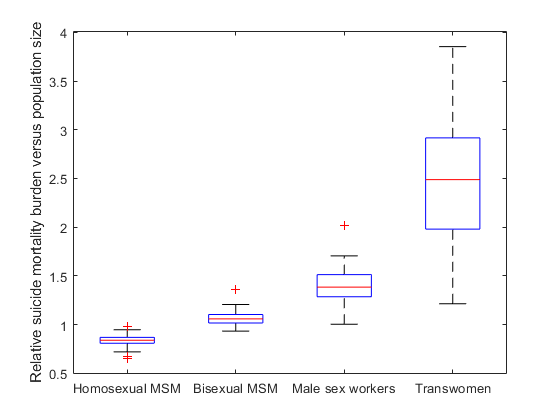
**

**PrEP prioritization by gender identity/sexual behaviour***.* We implemented a scenario in which 100% of TW would be covered, with the remainder given to MSW in order to compare the effectiveness of this established strategy to that of PrEP prioritization by stimulant use. In this scenario, we assumed the same total PrEP coverage as in the other two senarios (corresponding to the proportion of MSM/TW who use stimulants and are HIV negative) and covered 100% of HIV negative TW and allocated the remaining PrEO to MSW independently of stimulant use.

This PrEP prioritization strategy based on gender identity/sexual behaviour led to a 22% (95%I: 9-41%) reduction in new HIV infections between 2020-2030, which is 23% greater than obtained through the prioritization strategy based on stimulant use.

# References

1. Gomez GB, Borquez A, Caceres CF, et al. The potential impact of pre-exposure prophylaxis for HIV prevention among men who have sex with men and transwomen in Lima, Peru: a mathematical modelling study. *PLoS Med* 2012; **9**(10): e1001323.

2. Farrell M MN, Stockings E, Bórquez A, Cepeda J, Degenhardt L, Robert A, Tran L, Rehm J, Torrens M, Shoptaw S, McKetin M, . Responding to global stimulant use: challenges and opportunities. *Lancet (Under review)* 2019.

3. Hollingsworth TD, Anderson RM, Fraser C. HIV-1 transmission, by stage of infection. *J Infect Dis* 2008; **198**(5): 687-93.

4. INEI. Censo de la poblacion. 2007. <http://censos.inei.gob.pe/censos2007/>.

5. Sanchez J, Lama JR, Kusunoki L, et al. HIV-1, sexually transmitted infections, and sexual behavior trends among men who have sex with men in Lima, Peru. *J Acquir Immune Defic Syndr* 2007; **44**(5): 578-85.

6. Ley Nº 28243: Ley que amplia y modifica la Ley 266246 sobre el virus de inmunodeficiencia humana (VIH), el sindrome de inmunodeficiencia adquirida (SIDA) y las infecciones de transmision sexual. May 2004. <http://www.congreso.gob.pe/ntley/Imagenes/Leyes/28243.pdf>.

7. UNAIDS. Peru: Epidemiological Fact Sheet on HIV and AIDS: Core data on epidemiology and response 2008 update. <http://apps.who.int/globalatlas/predefinedReports/EFS2008/full/EFS2008_PE.pdf> (accessed.

8. Chow JY, Konda KA, Borquez A, et al. Peru's HIV care continuum among men who have sex with men and transgender women: opportunities to optimize treatment and prevention. *Int J STD AIDS* 2016; **27**(12): 1039-48.

9. McCormack S, Dunn DT, Desai M, et al. Pre-exposure prophylaxis to prevent the acquisition of HIV-1 infection (PROUD): effectiveness results from the pilot phase of a pragmatic open-label randomised trial. *Lancet* 2016; **387**(10013): 53-60.

10. Roux P, Fressard L, Suzan-Monti M, et al. Is on-Demand HIV Pre-exposure Prophylaxis a Suitable Tool for Men Who Have Sex With Men Who Practice Chemsex? Results From a Substudy of the ANRS-IPERGAY Trial. *J Acquir Immune Defic Syndr* 2018; **79**(2): e69-e75.

11. INEI. Censo de la poblacion. 1981. <http://www.inei.gob.pe/>.

12. Patrucco R. Síndrome de Inmunodeficiencia Adquirida en el Perú (Sida). Estudios Inmunológicos. *Diagnóstico* 1985; **16**(5): 122-35.

13. Caceres C, Konda K, Pecheny M, Chatterjee A, Lyerla R. Estimating the number of men who have sex with men in low and middle income countries. *Sex Transm Infect* 2006; **82 Suppl 3**: iii3-9.

14. Garcia P, Holmes KK, Garnett GP. The PREVEN Project: Urban community randomized trial for prevention of STI in Peru. Peru; 2006.

15. PAHO. Modos de Transmisión del VIH en América Latina: Resultados de la aplicación del modelo. Lima: MINSA, 2009.

16. Baggaley RF, Garnett GP, Ferguson NM. Modelling the impact of antiretroviral use in resource-poor settings. *PLoS Med* 2006; **3**(4): e124.

17. Baggaley RF, White RG, Boily MC. HIV transmission risk through anal intercourse: systematic review, meta-analysis and implications for HIV prevention. *Int J Epidemiol* 2010; **39**(4): 1048-63.

18. Vittinghoff E, Douglas J, Judson F, McKirnan D, MacQueen K, Buchbinder SP. Per-contact risk of human immunodeficiency virus transmission between male sexual partners. *American journal of epidemiology* 1999; **150**(3): 306-11.

19. Donnell D, Baeten JM, Kiarie J, et al. Heterosexual HIV-1 transmission after initiation of antiretroviral therapy: a prospective cohort analysis. *Lancet* 2010; **375**(9731): 2092-8.

20. UNAIDS. Informe Nacional de UNGASS, 2009.

21. Pinkerton SD, Abramson PR. Effectiveness of condoms in preventing HIV transmission. *Social science & medicine (1982)* 1997; **44**(9): 1303-12.

22. Weller S, Davis K. Condom effectiveness in reducing heterosexual HIV transmission. *Cochrane database of systematic reviews (Online)* 2002; (1): CD003255.

23. Caceres CF, Konda K, Segura ER, Lyerla R. Epidemiology of male same-sex behaviour and associated sexual health indicators in low- and middle-income countries: 2003-2007 estimates. *Sex Transm Infect* 2008; **84 Suppl 1**: i49-i56.

24. Lama JR, Lucchetti A, Suarez L, et al. Association of herpes simplex virus type 2 infection and syphilis with human immunodeficiency virus infection among men who have sex with men in Peru. *J Infect Dis* 2006; **194**(10): 1459-66.

25. Sanchez J, Lama JR, Peinado J, et al. High HIV and ulcerative sexually transmitted infection incidence estimates among men who have sex with men in Peru: awaiting for an effective preventive intervention. *J Acquir Immune Defic Syndr* 2009; **51 Suppl 1**: S47-51.

26. Caceres CF, Konda KA, Salazar X, et al. New populations at high risk of HIV/STIs in low-income, urban coastal Peru. *AIDS Behav* 2008; **12**(4): 544-51.

27. Clark JL, Caceres CF, Lescano AG, et al. Prevalence of same-sex sexual behavior and associated characteristics among low-income urban males in Peru. *PLoS One* 2007; **2**(8): e778.

28. Clark JL, Konda KA, Segura ER, et al. Risk factors for the spread of HIV and other sexually transmitted infections among men who have sex with men infected with HIV in Lima, Peru. *Sex Transm Infect* 2008; **84**(6): 449-54.

29. Stover J, Bertrand JT, Shelton JD. Empirically based conversion factors for calculating couple-years of protection. *Evaluation review* 2000; **24**(1): 3-46.

30. Konda KA, Lescano AG, Leontsini E, et al. High rates of sex with men among high-risk, heterosexually-identified men in low-income, coastal Peru. *AIDS Behav* 2008; **12**(3): 483-91.

31. Caceres C, Segura ER. Unpublished data: CPOS study - baseline data. 2011.

32. Caceres C, Segura ER. Unpublished data: Trans-Amfar study - baseline data. 2011.

33. Miller GA, Mendoza W, Krone MR, et al. Clients of female sex workers in Lima, Peru: a bridge population for sexually transmitted disease/HIV transmission? *Sexually transmitted diseases* 2004; **31**(6): 337-42.

34. Tabet S, Sanchez J, Lama J, et al. HIV, syphilis and heterosexual bridging among Peruvian men who have sex with men. *AIDS* 2002; **16**(9): 1271-7.

35. Goodreau SM, Goicochea LP, Sanchez J. Sexual role and transmission of HIV Type 1 among men who have sex with men, in Peru. *J Infect Dis* 2005; **191 Suppl 1**: S147-58.

36. Rojas G, Gotuzzo E, Yi A, Koster F. Acquired immunodeficiency syndrome in Peru. *Ann Intern Med* 1986; **105**(3): 465-6.

37. Caceres C, Gotuzzo E, Wignall S, Campos M. Sexual behavior and frequency of antibodies to type 1 human immunodeficiency virus (HIV-1) in a group of Peruvian male homosexuals. *Bull Pan Am Health Organ* 1991; **25**(4): 306-19.

38. McCarthy MC, Wignall FS, Sanchez J, et al. The epidemiology of HIV-1 infection in Peru, 1986-1990. *AIDS* 1996; **10**(10): 1141-5.

39. Calleja JM, Walker N, Cuchi P, Lazzari S, Ghys PD, Zacarias F. Status of the HIV/AIDS epidemic and methods to monitor it in the Latin America and Caribbean region. *AIDS* 2002; **16 Suppl 3**: S3-12.

40. Estudio de Vigilancia Epidemiológica de ITS y VIH en Hombres que Tienen Sexo con Hombres Comparando las Metodologías de Reclutamiento: Muestreo por Conveniencia, Muestreo por Tiempo y Espacio y el Muestreo Dirigido por Participantes. Lima, Peru: Coordinadora Nacional Multisectorial en Salud, Fondo Mundial de Lucha Contra el Sida, la Tuberculosis y la Malaria, CARE-Peru; Nov 11, 2011.

41. Best D, Noble A, Finch E, Gossop M, Sidwell C, Strang J. Accuracy of perceptions of hepatitis B and C status: cross sectional investigation of opiate addicts in treatment. *BMJ* 1999; **319**: 290 - 1.

42. Clark JL, C. M. Zigler, J. D. Klausner, et al,. Use of a Bed IGG Immunocapture Assay to Estimated Incidence of HIV and Associated Risk Factors in a High-Risk Population in Lima, Peru. International Society for Sexually Transmitted Diseases Research 17th Meeting. Seattle, Washington, USA; 2007.

43. Guanira J, Lama, P., Goicochea, et al.,. HIV Incidence and Syphilis Rates among Men Who Have Sex with Men at High Risk for HIV-1 Infection in 5 Andean Cities. 14th Conference on Retroviruses and Opportunistic Infections; 2007; Los Angeles, California, USA; 2007.

44. R. Castillo KAK, S.R. Leon, A. Silva-Santisteban, X. Salazar, T.J. Coates, C.F. Caceres,. HIV/STI incidence and associated risk factors among high-risk MSM and male-to-female transgender women in Lima, Peru. 20th International AIDS Conference. Melbourne, Australia; 2014.

45. Villaran M, A. Brezak, S. Ahmed, et al,. A Study of Potential HIV Transmission Hotspots among Men Who Have Sex with Men and Transgender Women in Lima, Peru. 21st International AIDS Conference. Durban, South Africa; 2016.

46. Goodman-Meza D, Beymer MR, Kofron RM, et al. Effective use of pre-exposure prophylaxis (PrEP) Among stimulant users with multiple condomless sex partners: a longitudinal study of men who have sex with men in Los Angeles. *AIDS Care* 2019: 1-6.

47. Hojilla JC, Satre DD, Glidden DV, et al. Brief Report: Cocaine Use and Pre-exposure Prophylaxis: Adherence, Care Engagement, and Kidney Function. *J Acquir Immune Defic Syndr* 2019; **81**(1): 78-82.

48. Hojilla JC, Vlahov D, Glidden DV, et al. Skating on thin ice: stimulant use and sub-optimal adherence to HIV pre-exposure prophylaxis. *J Int AIDS Soc* 2018; **21**(3): e25103.
